# Observation of super-Nernstian proton-coupled electron transfer and elucidation of nature of charge carriers in a multiredox conjugated polymer†

**DOI:** 10.1039/d4sc00785a

**Published:** 2024-04-22

**Authors:** Duyen K. Tran, Sarah M. West, Elizabeth M. K. Speck, Samson A. Jenekhe

**Affiliations:** a Department of Chemical Engineering, University of Washington Seattle Washington 98195-1750 USA jenekhe@uw.edu; b Department of Chemistry, University of Washington Seattle Washington 98195-1750 USA

## Abstract

Nernstian proton-coupled electron transfer (PCET) is a fundamental process central to many physical and biological systems, such as electrocatalysis, enzyme operation, DNA biosynthesis, pH-/bio-sensors, and electrochemical energy storage devices. We report herein the discovery of super-Nernstian PCET behavior with two protons per electron transferred in the electrochemical doping of a redox conjugated polymer, phenazine-substituted ladder poly(benzimidazobenzophenanthroline) (BBL-P), in aqueous electrolyte. We show that the super-Nernstian response originates from existence of multiredox centers that have a gradient of p*K*_a_ on the conjugated polymer. Our use of various pH-dependent *in operando* techniques to probe the nature of charge carriers in n-doped BBL-P found that polarons are the charge carriers at low to intermediate levels of doping (0.1–1.0 electron per repeat unit (eru)) whereas at higher doing levels (1.3 eru), polarons, polaron pairs, and bipolarons co-exist, which evolve into strongly coupled polaron pairs at the highest doping levels (>1.5 eru). We show that PCET-assisted n-doping of BBL-P results in very high redox capacity (>1200 F cm^−3^) in acidic electrolyte. Our results provide important new insights into PCET in organic materials and the nature of charge carriers in n-doped conjugated polymers while having implications for various electrochemical devices.

## Abstract 2

Observation of super-Nernstian proton-coupled electron transfer behavior with two protons per electron transferred in an electrochemically n-doped redox conjugated polymer.

## Graphical abstract

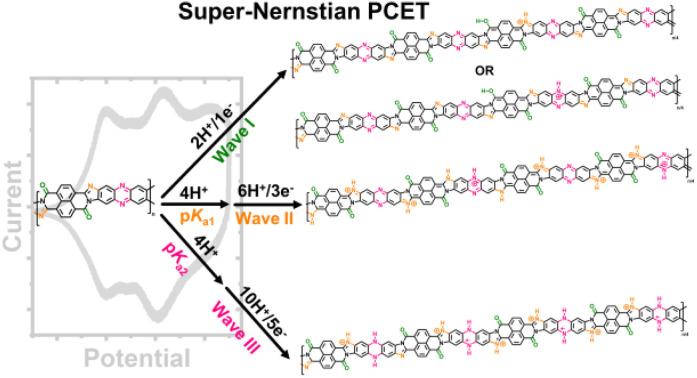

## Introduction

Proton-coupled electron transfer (PCET) is a fundamental process that lies at the heart of many physical and biological systems^1–6^ such as photosynthesis and respiration,^3–5^ DNA biosynthesis,^3^ enzyme operation,^5,6^ water splitting for fuel cells,^4^ nitrogen fixation,^3^ chemical sensors,^5^ electrochemical devices,^5^*etc.* Compared to the sole electron transfer, PCET has been shown to be a more thermodynamically favorable pathway as it effectively lowers the activation energy barriers between the reactants and the products.^7^ The majority of reported PCET processes feature stoichiometric proton/electron ratios as evidenced by a Nernstian shift (59.2 mV pH^−1^) in the Pourbaix diagram.^4,8^ Very few exceptions from the stoichiometric proton/electron ratios, or deviation from Nernstian behavior, have been observed.^4,9–14^ In particular, hydrated inorganic oxides with redox-active transition metal centers (*e.g.* nickel-borate, iridium, ruthenium) can afford a super-Nernstian shift (70–120 mV pH^−1^),^9–12^ whereby the additional proton uptake can be attributed to the presence of water molecules since thermal annealing of such oxides to rigorously remove excess moisture resulted in a conventional Nernstian shift.^10^ In addition to inorganic oxides, biological molecules such as iron-coordinating histidine based metalloprotein have also produced super-Nernstian shift^4,13,14^ due to its unique molecular structure which features separate electron sinks (*i.e.* ferrous/ferric redox centers) and proton sinks (*i.e.* histidine groups). These observed cases of multi-proton coupled electron transfers all require a transition metal center, which means that super-Nernstian PCET has yet to be discovered in an all-organic material. Fundamental questions, such as *is it possible to achieve super-Nernstian PCET without metal centers? What are the structural features that could facilitate super-Nernstian PCET in organic materials?* remain to be explored with potential implications for ultrasensitive pH sensing, enhanced electrochemical proton storage, proton batteries, and single-phase dual proton–electron conduction catalyst, among others.

**Chart 1:**
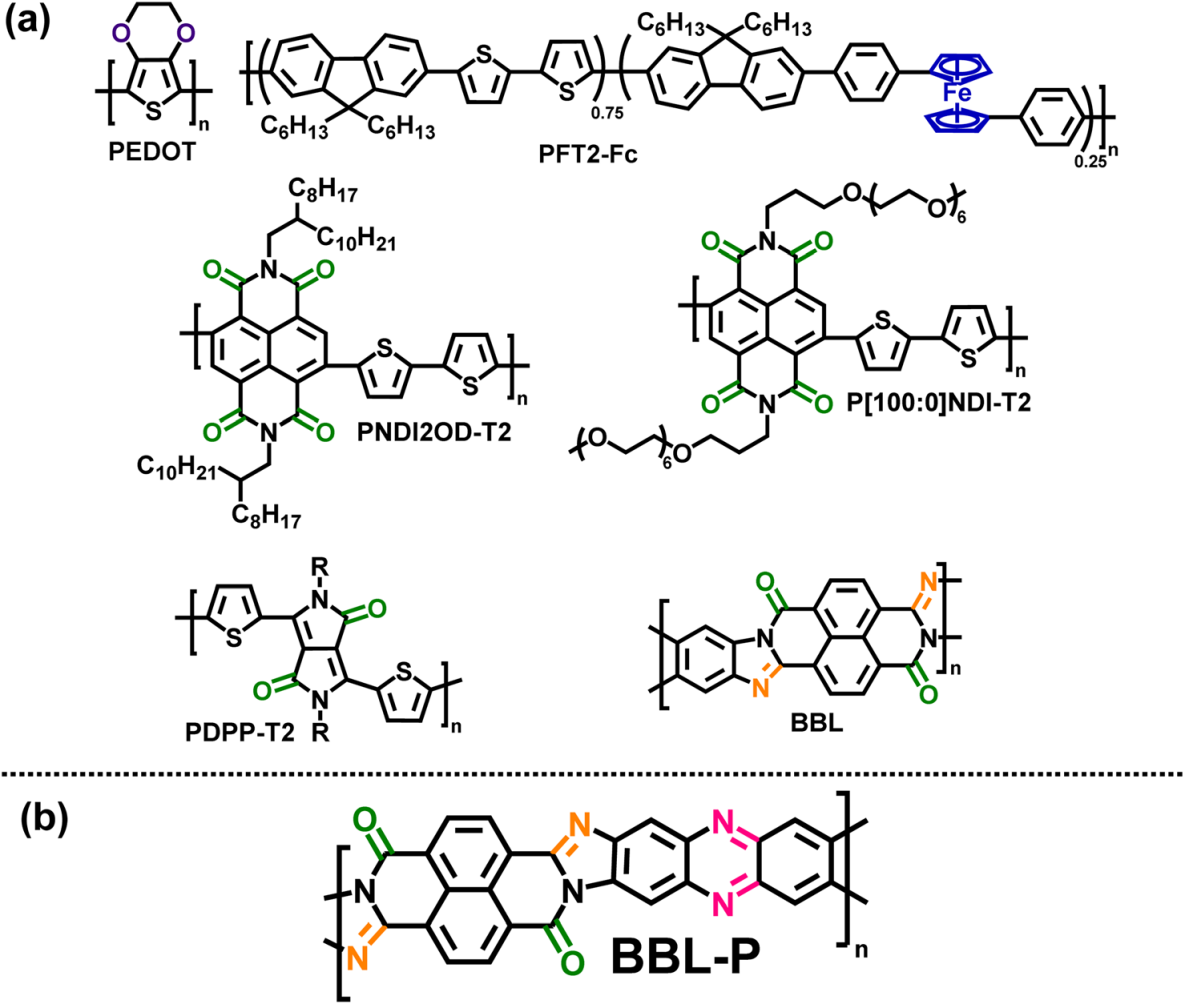
(a) Molecular structures of some previously reported redox π-conjugated polymers;^16,18,22,23,26,27^ (b) molecular structure of a multiredox π-conjugated polymer, BBL-P.

Redox π-conjugated polymers are an ideal platform to address the above research questions. Unlike nonconjugated redox polymers which typically contain pendant redox groups (*e.g.* quinone, imides, ketones, radicals, *etc.*),^15^ the class of redox π-conjugated polymers contain redox-active sites within the polymer backbone; thus, combining the robust charging/discharging nature of redox sites with the enhanced electronic charge delocalization and transport along the conjugated polymer backbone.^16–22^ Although traditional redox-active π-conjugated polymers are usually conducting polymers such as polyaniline (PANi), polypyrrole (PPy), or poly(3,4-ethylenedioxythiophene) (PEDOT) (Chart 1a)^23^ or metallopolymers^24^ (Chart 1a), current state-of-the-art redox π-conjugated polymers incorporate molecular redox sites into the backbone such as carbonyl group (–C=O–) containing polymers including naphthalene diimide (NDI) based copolymers^16–19^ (Chart 1a) and diketopyrrolopyrrole (DPP) based copolymers (Chart 1a).^21,22^ Although a detailed measurement and analysis of the Pourbaix diagram was absent in these previously reported redox π-conjugated polymers, they were assumed to follow the Nernstian response.^16–22^

The ability to store charges with excellent stability and robust reversibility in electrochemically n-doped redox π-conjugated polymers^16,25^ could also enable fundamental studies to explore the nature of charge carriers (*e.g.* electron-polarons, electron-bipolarons, *etc.*) in the materials. In fact, current understanding of the structure–electrochemical property-device performance relationships remain modest for n-type π-conjugated polymers, due in part to the polymer backbone unable to stabilize large amounts of injected electrons upon doping.^16^ As a result, there still exists a large knowledge gap regarding the electrochemical reactivity of the polymers and how tuning of such a material property influences the performance of electrochemical devices.

Here, we report use of a novel multiredox π-conjugated polymer, BBL-P (Chart 1b) to simultaneously investigate the PCET process and the nature of charge carriers in electrochemically n-doped BBL-P in aqueous electrolyte. The highly planar rigid-rod backbone of BBL-P facilitates large delocalization length that could stabilize the charged species upon formation, and the six total redox-active sites, consisting of two carbonyl groups and four imine sites (Chart 1b) can also facilitate multiple reversible and rapid electron injections. We hypothesize that the multiple redox sites of different p*K*_a_ values could enable a super-Nernstian PCET response when BBL-P is electrochemically reduced in aqueous electrolyte. Furthermore, a high doping level could be achieved, which will aid in distinguishing whether polaron pairs or bipolarons are the multiply charged species in heavily doped BBL-P. Furthermore, the more disordered nature of BBL-P^28^ will help to stabilize the formation of multiply charged species,^29,30^ which otherwise would be harder in the more crystalline parent polymer, BBL (Chart 1a).^31,32^

We found that BBL-P undergoes three acid-base coupled redox reactions corresponding to the different molecular redox sites with decreasing p*K*_a_ from naphthalene imine to phenazine imine to carbonyl oxygen. Each redox reaction is discovered to exhibit a super-Nernstian shift, involving two protons per one electron transferred, which is unusual and deviates from the commonly observed stoichiometric proton/electron ratio seen in most PCET processes.^7,8,33^ We similarly found that the electrochemical reduction of the parent polymer BBL (Chart 1a) thin films also show a super-Nernstian behavior albeit only 1.5 protons are coupled to every electron transferred. The observed super-Nernstian behavior is shown to originate from the multiredox centers with varying p*K*_a_ in the conjugated polymer backbone. By using various pH-dependent *in operando* techniques to probe the nature of charge carriers in electrochemically n-doped BBL-P, we show the evolution of polarons at low to intermediate levels of doping (0.1–1.0 eru) to an equilibrium mixture of polarons, polaron pairs, and bipolarons at a higher level of doping (1.3 eru) and finally to polaron pairs at the highest level of doping (≥1.5 eru). Finally, we show that PCET-assisted n-doping of BBL-P in acidic electrolyte leads to a very high redox capacity (>1200 F cm^−3^).

## Experimental methods

### Chemicals

The phenazine-substituted poly(benzimidazobenzophenanthroline) ladder polymer, BBL-P, was synthesized according to our previously reported procedure.^28^ Methanesulfonic acid (MSA) was purchased from Sigma-Aldrich and used as received.

### Preparation of BBL-P films on electrodes

The BBL-P sample of 1.1 dL g^−1^ intrinsic viscosity was dissolved in methanesulfonic acid (MSA; 15 mg mL^−1^) and stirred at elevated temperature (>100 °C) for several days. The polymer solution was filtered through 1 μm pore size Grade GF/B Glass Microfiber Filters (Whatman GF/B w/GMF) immediately before use. The filtered polymer solution was spin-coated at 1500 rpm for 30 seconds onto ITO substrates for cyclic voltammetry, onto FTO substrates for spectroelectrochemistry, and onto gold coated glass substrates for *in operando* Raman spectroscopy. The acidic solvent was immediately removed by immersing the polymer films into a mixture of isopropanol (IPA) and ethylene glycol (EG) (IPA : EG = 1 : 1, v : v) several times over 12 hours. The polymer films were then dried in a vacuum oven at 90 °C overnight and then annealed at 170 °C on a hot plate for 10 minutes in ambient conditions.

### Cyclic voltammetry

Cyclic voltammetry was performed in conventional three-electrode cells with Ag/AgCl pellet as the reference electrode (World Precision Instrument) and Pt mesh as the counter electrode. Cyclic voltammetry measurements were performed at room temperature using EG&G Princeton Applied Research, model 273A potentiostat. The BBL-P film prepared as described above served as the working electrode. The supporting electrolyte consisted of 0.1 M KCl in deionized (DI) water, and its pH values were tuned by adding either KOH or HCl such that the final solution always contained at least 0.1 M KCl. The 0.1 M KCl_(aq)_ electrolyte was purged with a nitrogen stream for at least 15 minutes before any measurements. A stream of N_2_ was also constantly flown on top of the electrolyte surface during measurements to minimize oxygen diffusion.

### Optical absorption and spectroelectrochemistry

Optical absorption spectra of the BBL-P films were taken on a PerkinElmer Lambda 900 spectrometer. For spectroelectrochemistry, BBL-P thin film was coated on FTO substrates which were inserted into a cuvette filled with 0.1 M KCl_(aq)_ as the electrolyte. The polymer film area was 2.75 ± 0.18 cm^2^, and the polymer film thickness was 44.4 ± 8.8 nm. Three-electrode configuration containing Ag/AgCl pellet as the reference electrode, Pt mesh as the counter electrode, and FTO/BBL-P as the working electrode was used. A Metrohm Autolab PGSTAT302N potentiostat was used to control the potential *via* the Metrohm NOVA software (Version 2.1.6). The BBL-P working electrodes were biased at different potentials for 60 s for doping to be equilibrated before collecting the optical absorption spectra. The films were thoroughly de-doped by applying +0.5 V (*vs.* Ag/Ag^+^) between each doping cycle. Both the doping and the de-doping currents were simultaneously collected during the optical measurements for coulometry analysis. The electrolyte was degassed by purging with N_2_ stream for at least 20 min prior to measurements.

### *In operando* Raman spectroscopy

Raman spectra of BBL-P thin films were collected using a Thermo Scientific DXR2 Raman microscope. A 532 nm laser with a power of 2 mW was focused onto BBL-P film surface through a 10× objective lens. *In operando* Raman spectra were taken at different bias potentials and different pH values. A three-electrode cell consisting of BBL-P on gold coated glass substrate as the working electrode, Pt mesh as the counter electrode, and Ag/AgCl pellet as the reference electrode, and a Metrohm Autolab PGSTAT302N potentiostat was used to control the potential *via* the Metrohm NOVA software (Version 2.1.6).

## Results and discussion

### pH-dependent cyclic voltammetry of BBL-P in aqueous electrolyte

**Fig. 1:**
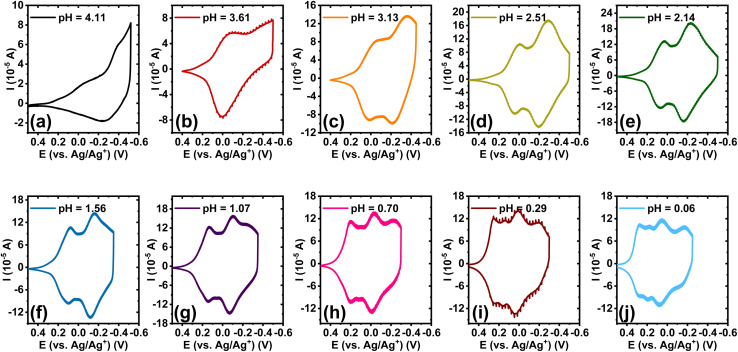
pH-dependent cyclic voltammograms of BBL-P thin films collected in 0.1 M KCl_(aq)_ supporting electrolyte with a scan rate of 25 mV s^−1^ by using a 3-electrode configuration with ITO/BBL-P as the working electrode, Pt mesh as the counter electrode, and Ag/AgCl as the reference electrode: (a) pH = 4.11; (b) pH = 3.61; (c) pH = 3.13; (d) pH = 2.51; (e) pH = 2.14; (f) pH = 1.56; (g) pH = 1.07; (h) pH = 0.70; (i) pH = 0.29; and (j) pH = 0.06.

Cyclic voltammetry (CV) was performed to investigate the electrochemical properties of BBL-P in an aqueous environment. A three-electrode electrochemical cell, consisting of a Ag/AgCl pellet reference electrode, Pt mesh as the counter electrode, and BBL-P thin film coated on a conducting ITO substrate as the working electrode, was used. The aqueous electrolyte always contained at least 0.1 M KCl_(aq),_ and the pH of the electrolyte solution was varied by adding either hydrochloric acid (HCl) or potassium hydroxide (KOH). Additionally, acetonitrile was also titrated to the electrolyte at 10 vol% to poison the Pt electrode and suppress any hydrogen evolution reaction. The applied potentials were also kept within the stability window of water as a function of pH to ensure that no undesirable side reactions took place. The final electrolyte solution was de-oxygenated by purging with nitrogen (N_2_) for 20 minutes before any measurements, and a constant N_2_ stream was also maintained atop of the electrolyte surface to ensure an oxygen-free environment during the measurements. Cyclic voltammograms of BBL-P thin films with varying electrolyte's pH from pH = 4.11 to pH = 0.06 are shown in Fig. 1. Based on the number of observable features in the voltammograms, the pH-dependent electrochemical properties of BBL-P can be categorized into three separate regions:

#### Region 1

In the case of 3.1 < pH ≤ 8.1, the voltametric patterns showed marginal dependence on the electrolyte's pH (Fig. 1a and S1†). Indeed, only one voltametric feature, wave I, with poorly resolved cathodic and anodic waves can be observed with a formal potential (*E*_1/2_) of −0.32 V (*vs.* Ag/Ag^+^) and significant peak splitting (Δ*E*_p_) of 138.8 ± 5.8 mV. We note that the formal potential is the average potential of the potentials at which the reduction current and the oxidation current are maximized^34^ (Fig. S2†). As the pH value decreased to ∼3.6 (Fig. 1b), both the forward and return waves became more apparent with peak positions shifted towards more positive potentials with *E*_1/2_ of −0.037 V (*vs.* Ag/Ag^+^) and a smaller peak splitting (Δ*E*_p_ = 75.6 ± 11.5 mV at 25 mV s^−1^).

#### Region 2

In the case of 1 < pH < 3.1, the voltammograms showed two pairs of well-resolved and quasi-reversible reduction and oxidation waves, consisting of the previously observed wave I and an emerging wave II at more negative potentials (Fig. 1c–g). Both the formal potential and the peak splitting of wave I and wave II were found to strongly depend on the electrolyte's pH. In particular, the *E*_1/2_ value of wave I gradually shifted from −0.017 V (*vs.* Ag/Ag^+^) to 0.14 V (*vs.* Ag/Ag^+^) whereas that of wave II moved from −0.29 V (*vs.* Ag/Ag^+^) to −0.09 V (*vs.* Ag/Ag^+^) as the solution pH changed from 3.13 to 1.07. Furthermore, the Δ*E*_p_ value of wave I was found to decrease from 58.4 mV to 8.44 mV while that of wave II was found to diminish from 143.4 mV to 38.4 mV as the solution pH was lowered. Brilliant changes in the film color were clearly visible during the scans with excellent stability and reversibility under anaerobic conditions. The first reduction process showed minimal color changes; however, the second reduction process changed the initially lavender violet film to become gray color.

#### Region 3

In the case of pH < 1, the voltammograms exhibited three sets of well-resolved and quasi-reversible waves consisting of the previously observed waves I and II and an emerging wave III at the most negative potential. Similar to the voltametric behavior seen in region 2, the *E*_1/2_ and Δ*E*_p_ of all three waves showed intense pH dependence, whereby the formal potentials progressively shifted positively with greatly reduced peak splitting. Robust electrochromic effects were also observed in this region whereby the third reduction process changed the color of the film from gray to bright green. The presence of three reversible electrochemical activity indicates that BBL-P can undergo three proton-coupled electron transfer events in the current pH range of interests, which is more than that of the parent BBL ladder polymer.^26^ Considering the molecular structures of BBL and BBL-P (Chart 1), the additional redox reaction at pH < 1 must originate from the phenazine unit in BBL-P.

### Proton-coupled electron transfer (PCET) reactions in BBL-P

The above pH-dependent CV results suggest that BBL-P can undergo two acid-base equilibria in aqueous KCl solution with an average p*K*_a_ of 2.5 and 1, which correspond respectively to the protonation at the naphthalene imine site (HBBL-P^+^) and the phenazine imine site (HBBL-P^+^’). Indeed, the parent BBL polymer has been shown to exhibit a p*K*_a_ around 2.2 for the naphthalene imine site in aqueous tetrabutylammonium sulfate,^26^ whereas phenazine small molecules are known to undergo protonation with a p*K*_a_ around 1.2.^35,36^

**Fig. 2:**
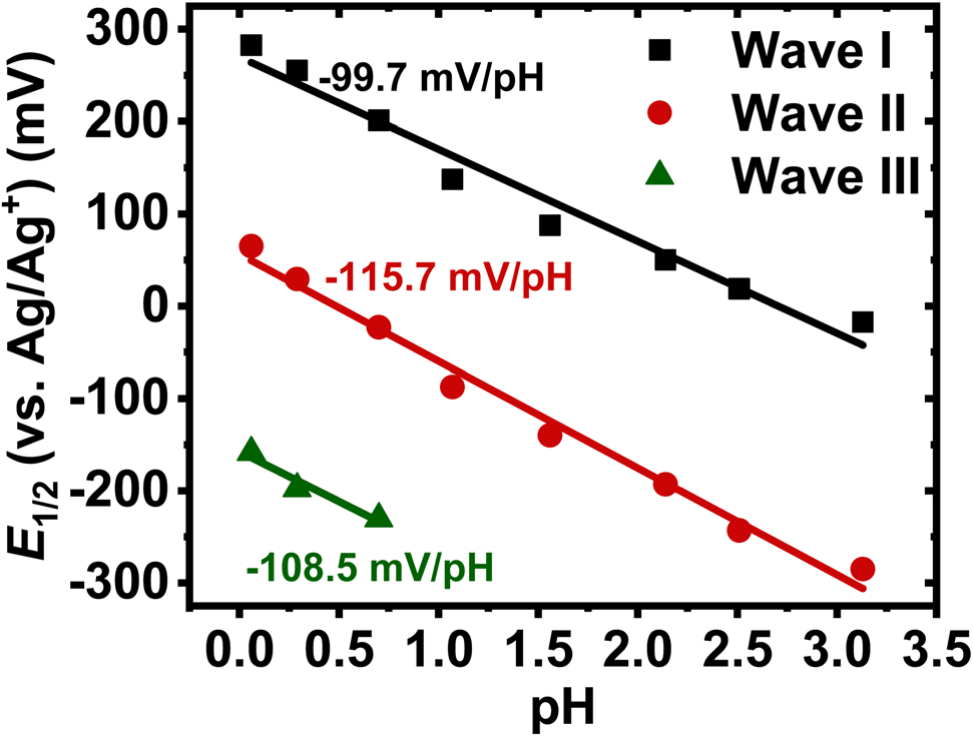
Representative Pourbaix diagram of BBL-P thin films in 0.1 M_(aq)_ KCl at a scan rate of 25 mV s^−1^.

Quantitative analysis of the pH-dependent formal potentials of each redox event *via* Pourbaix diagram (Fig. 2) revealed average slopes of −99.3 ± 0.8, −116.3 ± 1.1, and −109.6 ± 6.8 mV pH^−1^ for the first, second, and third redox reaction, respectively (Table 1). These results (|slope| > 59.2 mV pH^−1^) imply that BBL-P exhibits a super-Nernstian PCET behavior, where each redox reaction of BBL-P involves on average two protons per one electron (2H^+^/1e^−^). The numbers of protons per electrons (*n*_H+_/*n*_e−_) involved in each reduction event of BBL-P were also found to be independent of scan rates (Table 1). The observed super-Nernstian (|slope| > 59.2 mV pH^−1^) behavior of BBL-P, which deviates from the commonly observed one-proton/one-electron redox process, is very intriguing and worth further investigation. We conducted a similar experiment on the parent polymer BBL thin films, and the resulting cyclic voltammograms in aqueous electrolyte at various pH values are shown in Fig. S3.† Quantitative analysis of the Pourbaix diagram of BBL thin films (Fig. S4†) revealed average slopes of −89.2 ± 5.2 for the first redox process and −99.8 ± 3.9 mV pH^−1^ for the second redox process (Table S1†). These results thus suggest that BBL also exhibits a super-Nernstian behavior, featuring on average 1.5–1.7 protons per electron transferred in each reduction reaction. We note that in a previous attempt to understand the electrochemicaly doping reactions of BBL in aqueous electrolyte, a slightly smaller absolute value of slope of 78 mV pH^−1^ was observed, which corresponds to a *n*_H+_/*n*_e−_ value of about 1.3.^26^ However, in that prior report, the process was thought to follow Nernstian response to the first approximation;^26^ thus, overlooking any possibility of multi-proton coupled electron transfer. The observed higher *n*_H+_/*n*_e−_ of BBL thin film in our current study, which can be rationalized by subtle differences in film thickness, electrolytes, and polymer molecular weight, challenges the previously proposed PCET model of BBL.

**Table 1:** Quantification of proton-coupled electron transfer processes of BBL-P thin films at various scan rates

Scan rate (mV s^−1^)	Wave	Slope^a^ (mV pH^−1^)	*n*_H+_/*n*_e−_b
25	I	−99.3 ± 0.8	1.7 ± 0.01
25	II	−116.3 ± 1.1	2.0 ± 0.02
25	III	−109.6 ± 6.8	2.0 ± 0.10
50	I	−97.8 ± 1.1	1.7 ± 0.02
50	II	−115.1 ± 1.5	1.9 ± 0.02
50	III	−116.9 ± 4.1	2.0 ± 0.10
100	I	−97.3 ± 5.1	1.6 ± 0.10
100	II	−113.3 ± 0.7	1.9 ± 0.01
100	III	−119.3 ± 8.1	2.0 ± 0.14
150	I	−100.1 ± 13.9	1.7 ± 0.21
150	II	−116.1 ± 8.0	2.0 ± 0.02
150	III	−119.5 ± 3.2	2.0 ± 0.1

a Slope, average slope calculated from Pourbaix diagram (+/− one standard deviation).

b *n*_H+_/*n*_e−_, number of protons per electrons transferred in each reduction event of BBL-P (+/− one standard deviation). Average values and standard deviations were calculated from 5 different samples.

Our results show that BBL-P as well as its parent ladder polymer BBL exhibit super-Nernstian PCET processes upon electrochemical doping, which is very interesting and has yet to be reported for redox polymers.^15,25,37^ We hypothesize that the super-Nernstian PCET response seen in BBL-P originates from its unique molecular structure, which features multiple protonatable redox sites of different p*K*_a_ values in the polymer backbone. In particular, upon reduction of BBL-P at the carbonyl position, the electron density around both the naphthalene imine and the phenazine imine sites would increase owing to strong delocalization of π-electrons. As a result, the p*K*_a_ values of both imine redox sites would be higher in the reduced states than those in the neutral state, which means that they are more susceptible to protonation with consequent observed super-Nernstian PCET behavior. It is important to note that small shifts of the p*K*_a_ values of multiple protonatable sites in a molecular system is possible and has been previously reported in biologically relevant molecules, including Rieske iron-sulfur “charged ladder” metalloproteins.^38^

**Scheme 1:**
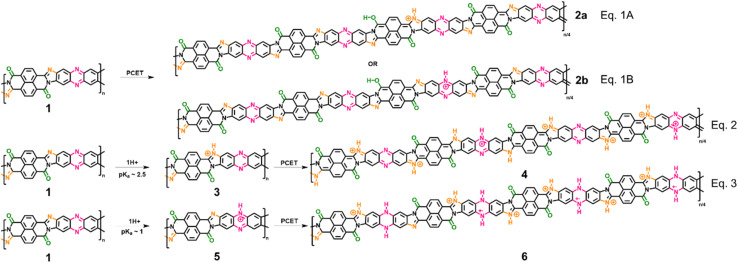
Proposed electrochemical reduction reactions of BBL-P in aqueous KCl electrolyte.

To confirm our hypothesis and arrive at a comprehensive mechanism underlying the observed super-Nernstian PCET-assisted electrochemical doping in BBL-P, we examined the thermodynamics and kinetics of each reduction process by density functional theory (DFT) calculations and variable scan rate cyclic voltammetry. Results from these analyses will provide macroscopic insights on the feasibility of the proposed mechanism. Furthermore, a combination of characterization techniques such as spectroelectrochemistry, coulometry, and *in operando* Raman spectroscopy were used to probe the evolution of optoelectronic properties, doping level, and molecular structure, all of which are discussed in detailed below to provide molecular insights into the electrochemical reduction processes. We propose that the extra proton involved in each electrochemical event of BBL-P is associated with the imine sites either on the naphthalene ring or on the phenazine unit, and the proposed multiple electrochemical reduction reactions of BBL-P in aqueous acidic electrolyte is shown in Scheme 1.

In the case of 3.1 < pH ≤ 8.1, the single redox wave (wave I) can be ascribed to the PCET reaction of BBL-P at the carbonyl oxygen as demonstrated in eqn 1A or 1B. As previously discussed, two pairs of well-resolved redox waves (wave I and wave II) were observed when 1 < pH < 3.1 (Fig. 1c–g). This result means that the reduced products of both reactions coexist in equilibrium whereby wave I corresponds to eqn 1A or 1B (Scheme 1), and wave II corresponds to the PCET reaction of BBL-P at the naphthalene imine site as shown in eqn 2 (Scheme 1). Similarly, when the pH of the electrolyte is less than 1, three pairs of pronounced and quasi-reversible redox waves, namely wave I, wave II, and wave III, were observed (Fig. 1h–g). In this case, three different reduced states of BBL-P coexist in equilibrium whereby wave I corresponds to eqn 1A or 1B (Scheme 1), wave II corresponds to eqn 2 (Scheme 1), and wave III corresponds to the reduction of BBL-P at the phenazine imine sites as demonstrated in eqn 3 (Scheme 1).

We emphasize that each reduction reaction is independent of one another and that the three redox waves are unlikely to be the sequential reduction reactions of BBL-P mainly due to the large energetic barrier required to inject additional electrons into the polymers upon the initial reduction. This phenomenon is in fact commonly observed for various organic redox molecules (M) where the potential of the second reduction reaction (Ṁ^−^ → M^2−^) are more negative than that of the first reduction reaction (M → Ṁ^−^) (Table S2†), and the potential difference (Δ*E*) are relatively large of around 0.4–0.7 V (Table S2†). In our case, the Δ*E* values were approximately 0.2–0.3 V, which is much smaller than the commonly observed Δ*E* of redox small molecules and polymers. Therefore, we assigned each reduction wave to an independent reduction reaction of BBL-P at different redox sites as outlined in Scheme 1.

### Thermodynamics of electrochemical doping reactions of BBL-P

We first examined the feasibility of the proposed electrochemical redox reactions of BBL-P from a thermodynamics perspective. Density functional theory (ωB97XD/6-31G (d,p)) of the dimers of the proposed structures (1–6) were performed to gain insights into the polymer chain conformations as well as their free energy. The optimized geometries of 1, 2, 4, and 6 structures all exhibited completely planar chain conformation (Fig. S5†), suggesting that the formation of polaron would not introduce any detrimental torsional defects into the rigid-rod polymer backbone.

A quantitative comparison of the driving force for each of the reduction reaction is presented in Fig. S6.† The Gibbs free energy change (Δ*G*) of all three reactions was found to be negative indicating that the reduced products (2, 4, and 6) are thermodynamically favorable. Furthermore, the Δ*G* value was largest for the 1 → 2 reaction (Δ*G*_12_ = −84 eV), which means that the conversion from neutral BBL-P to its reduced unprotonated form (eqn 1) is readily accessible; thus, requiring a low doping potential. The Δ*G* value of the 1 → 4 reaction was intermediate (Δ*G*_14_ = −67 eV), implying that the observed second reduction wave corresponds to the reduction of protonated BBL-P at the naphthalene imine sites (eqn 2). Moreover, the smallest Δ*G* value (Δ*G* = −66 eV) that corresponds to the 1 → 6 conversion suggests that protonated BBL-P at the phenazine imine sites (eqn 3) would be slightly harder to reduce and thus require substantially more negative potentials than reduction of the protonated naphthalene imine. These findings are in excellent agreement with the experimental CV data and have confirmed that the proposed electrochemical reduction reaction scheme of BBL-P is valid from a thermodynamic standpoint.

Furthermore, we examined the DFT computed atomic charge distribution of BBL-P dimers in different protonated and reduced states (Tables S3 and S4†) to test our hypothesis of p*K*_a_ shifts upon each PCET event. The electron density around both the naphthalene imine and phenazine imine was found to increase upon PCET; thus, making them susceptible to additional protonation. In particular, when the carbonyl oxygen underwent a 1H^+^/1e^−^ transfer, the electron density at the naphthalene imine sites (N45, N17) as well as the phenazine imine sites (N82, N70) increased (Table S3†). The larger electron density at these sites is indicative of enhanced basicity and higher p*K*_a_ values. As a result, these imine sites can be readily protonated, leading to the observed non-stoichiometric PCET. Similar phenomenon is also observed when the naphthalene imine nitrogen or phenazine imine nitrogen undergoes PCET-assisted electrochemical doping (Table S4†). For example, when the naphthalene imine undergoes a 1H^+^/1e^−^ transfer, the electron density at the phenazine imine sites (N81, N82) increased as evidenced by the more negative atomic charge distribution (Table S4†). This result suggests that the p*K*_a_ of these phenazine imine sites are higher compared to those in the neutral undoped BBL-P; thus, making them more susceptible to spontaneous protonation when 1 < pH < 3.

### Kinetics of electrochemical reduction reactions of BBL-P

**Fig. 3:**
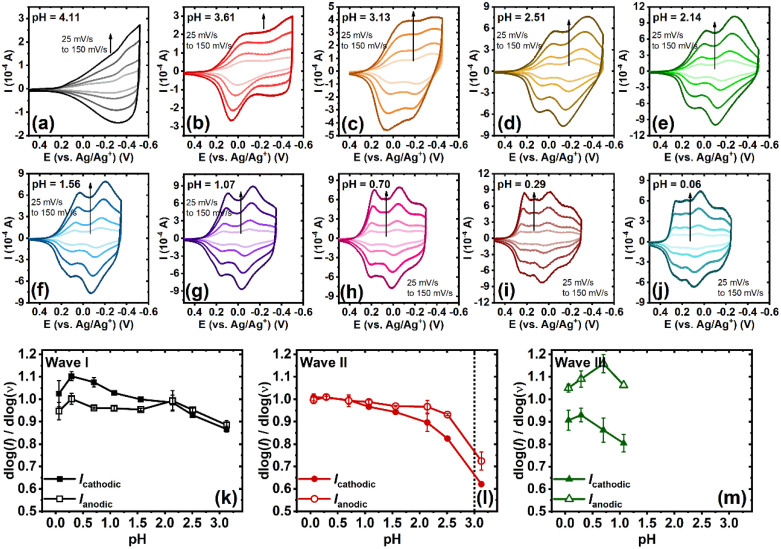
(a–j) pH-dependent cyclic voltammograms of BBL-P thin films collected in 0.1 M KCl_(aq)_ supporting electrolyte at various scan rates from 25 mV s^−1^ to 150 mV s^−1^. (k–m) Dependence of *b*-value 
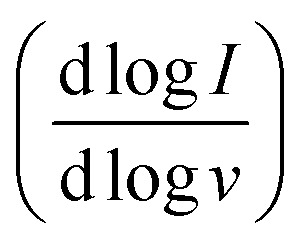
 on the electrolyte pH for the first redox event (k), the second redox event (l), and the third redox event (m).

The kinetics of each electrochemical event of BBL-P was also investigated to quantitatively distinguish between the slow diffusion-limited and the fast surface-controlled processes. Representative cyclic voltammograms of BBL-P with varying scan rate (*ν*) collected in electrolyte solution of pH ranging from 4.1 to 0.06 are shown in Fig. 3a–j. Notably, the voltametric responses of BBL-P showed marginal changes with increasing scan rate, showing well-resolved cathodic and anodic peaks up to 150 mV s^−1^ at all pH values. A power law is applied to describe the electrochemical reduction kinetics:*i* = *av*^*b*^where *i* is the current, *ν* is the scan rate, *a* is a constant, and *b* is the power-law coefficient.

The relationship between log(*i*) and log(*ν*) with varying electrolyte solution pH are shown in Fig. S7,† whereby the slope of the linear fit represents the *b*-value. The pH-dependence of *b*-value for each electrochemical reduction event is presented in Fig. 3k–m. In general, a *b*-value of 1 indicates a rapid surface-controlled pseudocapacitive electrochemical process whereas a *b*-value of 0.5 represents a battery-like slow diffusion-limited process.^39,40^ In the case of the first reduction event, average *b*-values of both the cathodic peak and the anodic peak were found to gradually increase from *b* = 0.87–0.89 to near unity (*b* = 1.00) as the pH values were lowered from 3.1 to 0.06 (Fig. 3k). This result suggests that the reduction of unprotonated BBL-P showed a dominant pseudocapacitive behavior, especially in highly acidic electrolytes. In the case of the second reduction process, the *b*-value exhibited a steep increase from around 0.6–0.7 to 0.9–1.0 as the solution pH decreased from ∼3 to ∼2.5 (Fig. 3l), which indicates a transition from a slow diffusion-limited process to a fast surface-limited redox process and coincides with the transition from CV region 1 to region 2 discussed earlier. The charge storage mechanism remained pseudocapacitive with decreasing pH down to 0.06 as evidenced by the relatively constant *b*-value around 1.0 (Fig. 3l). The kinetics of the third reduction process also showed a dominant pseudocapacitive behavior with average *b*-value around 0.9–1.1 regardless of the electrolyte solution pH (Fig. 3m). These results collectively suggest that the kinetics of electrochemical reduction of BBL-P can be controlled by the electrolyte's pH where protonation of the polymer offers an effective means to overcome the kinetics barrier, leading to rapid reduction processes. Furthermore, the charge storage mechanism of BBL-P follows a pseudocapacitive behavior even at high scan rates, which indicates the remarkable rate capabilities of BBL-P.^41^ Therefore, ladder polymer BBL-P could be a promising electrode material for future applications in all-polymer proton supercapacitors^42–44^ or proton batteries.^45–48^

We have thus far demonstrated that the proposed electrochemical reduction reactions of BBL-P are valid from macroscopic thermodynamic and kinetic perspectives. To further our understanding of the multiple redox states of BBL-P from a molecular level, we employed a complement of *in operando* characterization techniques as discussed in the following sections.

### Spectroelectrochemistry of BBL-P films in aqueous solution

We used spectroelectrochemistry to examine the evolution of optoelectronic properties upon electrochemical reduction as well as to probe the formation of polarons and bipolarons. The optical absorption spectra of undoped BBL-P with varying electrolyte's pH are shown in Fig. S8a.† Even when BBL-P is protonated (pH < 3 and pH < 1), the spectral shapes remain relatively constant with a main absorption feature centered at around 490–495 nm and a poorly resolved shoulder at around 630 nm. This result suggests that neither the electronic structures nor the polymer chain conformation were affected upon protonation, which is a unique characteristic of conjugated rigid-rod ladder polymers.^49^

**Fig. 4:**
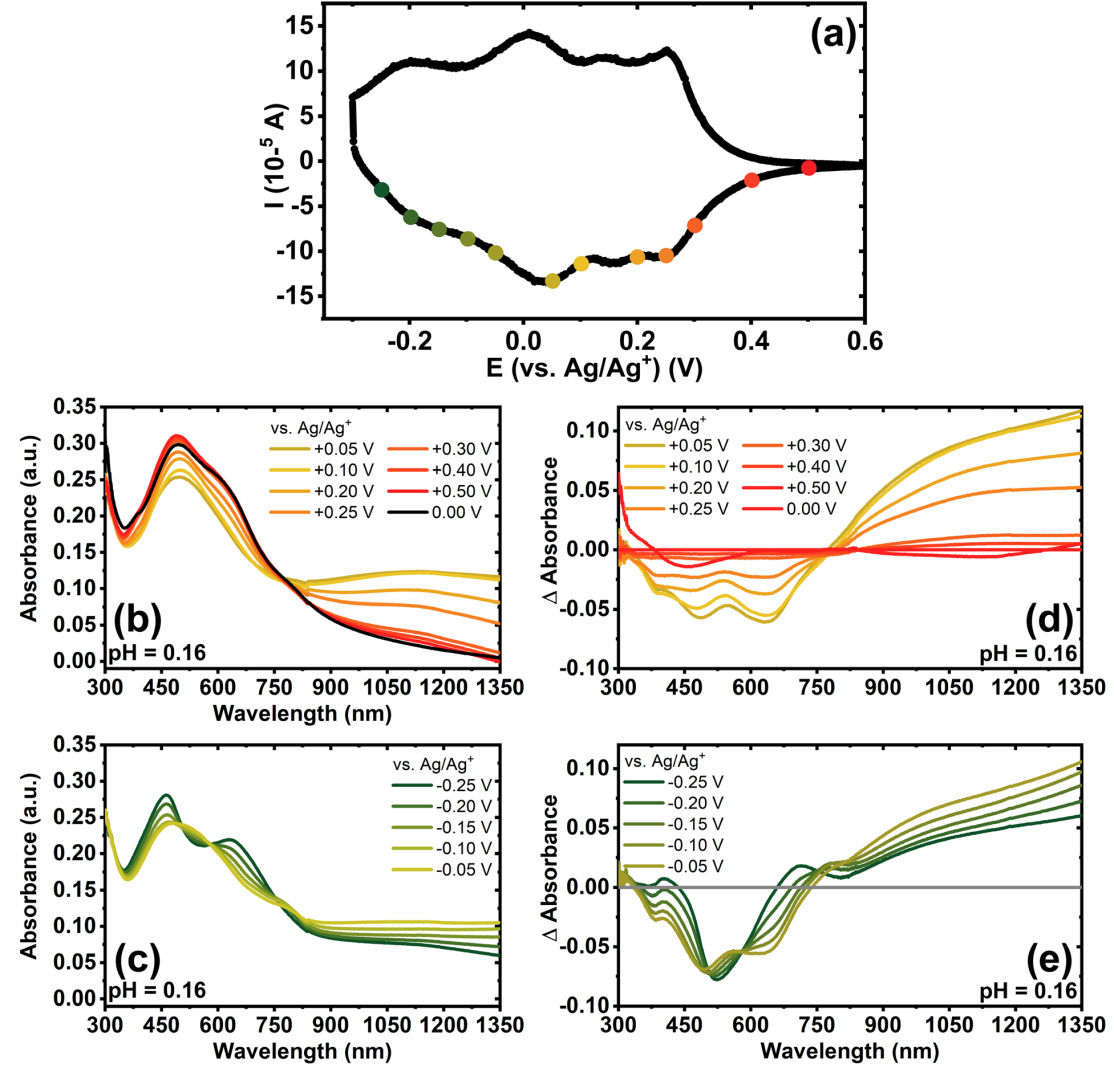
(a) Cyclic voltammogram of BBL-P thin films in aqueous KCl electrolyte of pH 0.29 collected at a scan rate of 25 mV s^−1^; (b and c) UV-Vis-NIR optical absorption spectra under different potentials of BBL-P thin films in electrolyte of pH = 0.16; (d and e) differential UV-Vis-NIR optical absorption spectra under different potentials of BBL-P thin films. The baseline spectrum was taken at +0.50 V to de-dope the films prior to each doping step. Note that spectrum labeled 0 V corresponds to absorption of the polymer films when submerged in the electrolyte without any applied potential.

Spectroelectrochemistry of BBL-P was performed in a three-electrode configuration with BBL-P coated on conducting transparent substrate as the working electrode, Pt mesh as the counter electrode, and Ag/AgCl pellet as the reference electrode. The optical properties of BBL-P thin films at various doping potentials collected in aqueous KCl electrolyte solution of pH 0.16, 1.04, and 2.46 are shown in Fig. 4, S9, and S10,† respectively. The points on the voltammograms (Fig. 4a, S9a, and S10a†) indicate the potentials at which the films were doped before collecting the spectra. The current was monitored to ensure that the polymer doping has reached a steady state before optical measurements took place. We note that the polymer films were thoroughly de-doped at +0.50 V (*vs.* Ag/Ag^+^) between each doping step. In each case, the potential was applied from negative to positive direction. The differential absorption spectra of BBL-P thin films as a function of doping potential and solution pH are also shown in Fig. 4d, e, S9d, e, and S10d, e.† Since results obtained in electrolyte solution of pH 1.04 and 2.46 (Fig. S9 and S10†) are essentially snapshots of those collected in extremely low pH region, we will focus our discussion on the spectroelectrochemistry of BBL-P in highly acidic electrolyte solution (pH < 1) where all three redox reactions coexist in equilibrium (eqn 1–3).

The spectral responses of BBL-P in highly acidic electrolyte (pH = 0.16) can be divided into two potential ranges as illustrated in Fig. 4b and c. As the applied potential approached the first redox peak at around +0.25 V (*vs.* Ag/Ag^+^) (Fig. 4a) from +0.50 V, the π–π* transition of the ground state (*λ*_max_ = 496 nm) decreased in intensity accompanied by a new broad absorption band in the near infrared (near-IR) region centered at 1115 nm (Fig. 4b and d), which indicates the formation of polarons. As the applied potential was swept towards the second redox event (*E* = +0.05 V, *vs.* Ag/Ag^+^), a combination of progressive increase of the near-IR absorption band (*λ* = 1115 nm) and gradual bleaching of the visible absorption band (*λ* = 496 nm) was observed (Fig. 4b and d), which means that the density of polarons increased with increasing doping level. Within this potential range from +0.50 V to +0.05 V (*vs.* Ag/Ag^+^), only one isosbestic point at around 770 nm (Fig. 4b and d) can be observed, indicating the direct conversion of neutral to the singly charged state of BBL-P. Furthermore, the density of polarons was found to be maximized at the second redox event (*E* = +0.05 V, *vs.* Ag/Ag^+^) as evidenced by the highest intensity of the near-IR band (Fig. 4d and S11a†). The differential absorption spectra also suggests that the density of polarons at the first redox event is about 2–2.5 times lower than that of the second redox event (Fig. S11a†).

As the applied potential approached the third redox event (*E* < 0 V, *vs.* Ag/Ag^+^), the π–π* transition of the ground state (*λ*_max_ = 496 nm) was significantly bleached giving rise to two sharp absorption bands centered at 461 nm and 640 nm (Fig. S11b†). The intensity of the near-IR absorption band (*λ* = 1115 nm) was also found to decrease from its maximum (Fig. S11a†), which suggests the transition of singly charged polarons to multiply charged species. Indeed, three isosbestic points at 505 nm, 580 nm, and 755 nm (Fig. 4c and e) can be seen indicating the conversion of singly charged to multiply charged states of BBL-P. Thus, the results show that the visible absorption bands at 461 nm and 640 nm can be assigned to the absorption of multiply charged BBL-P whereas the near-IR absorption band at 1115 nm is associated with the singly charged BBL-P. The extremely broad absorption features of BBL-P seen at applied potentials of −0.15 V to −0.05 V (*vs.* Ag/Ag^+^) (Fig. 4c) can be explained by the coexistence of both singly and multiply charged states, leading to an overlapping of several absorption features; thus, distorting the optical absorption spectra. As a result, both singly charged as well as multiply charged species can be charge carriers in the third redox event of BBL-P (*E*_1/2_ = −0.17 V, *vs.* Ag/Ag^+^).

### Coulometry of BBL-P films as a function of pH

**Fig. 5:**
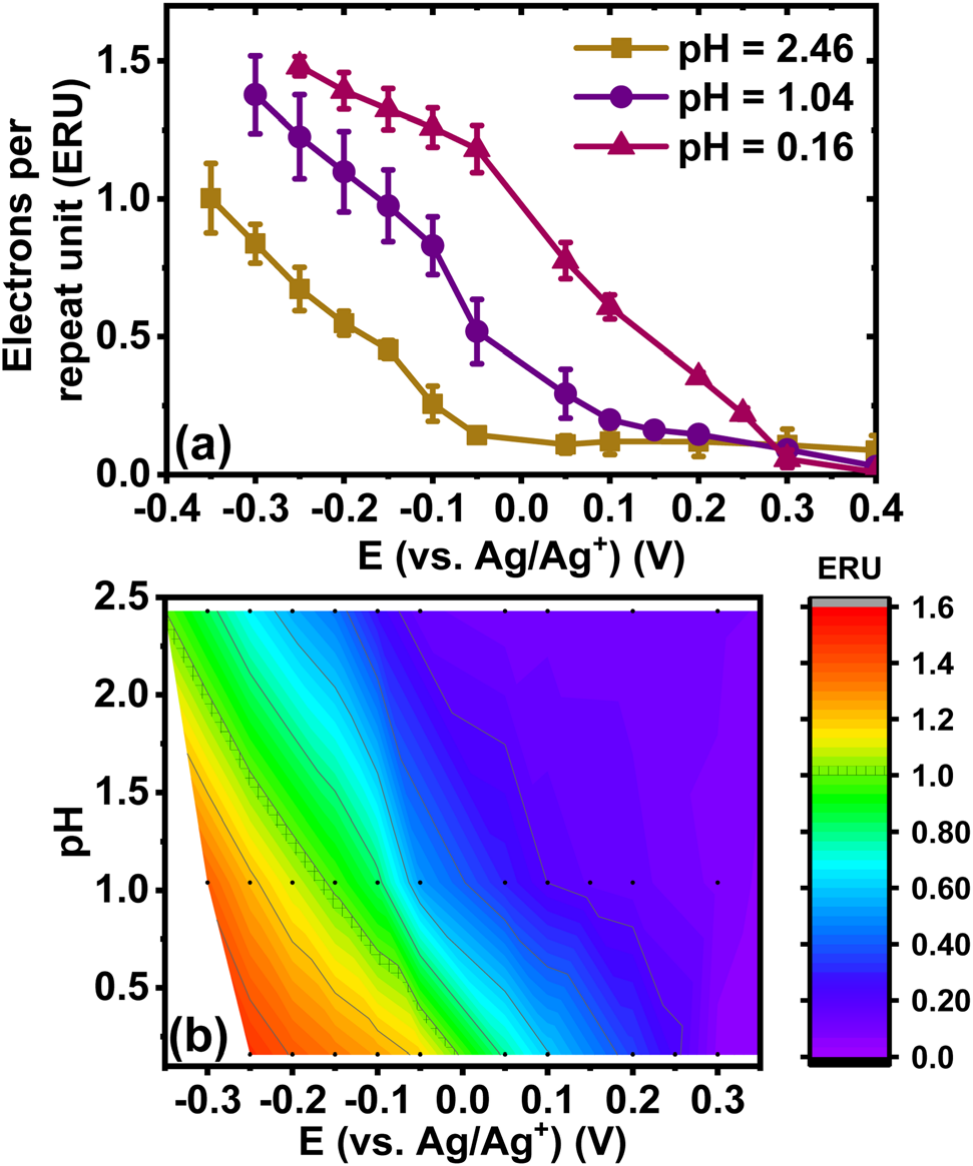
(a) Evolution of doping level (number of electrons per repeat unit) as a function of doping potential. (b) Dependence of doping level on electrolyte pH and doping potentials.

Next, we quantified the doping level by calculating the number of electrons per repeat unit (eru) *via* coulometry assay, which would also enable us to also verify the spectroelectrochemistry results. To avoid the effects of oxygen reduction reaction (ORR) and overestimation of charge integration, we used the de-doping current to calculate the electron density in the reduced BBL-P films after the optical absorption measurements.^50^ The number of electrons per repeat unit was calculated by normalizing the total charge generated in the reduced BBL-P films obtained by integration of the de-doping current to the number of monomers present on the electrode by following a previously reported method.^51^ The pH-dependence of the doping level is shown in Fig. 5a.

In highly acidic electrolyte (pH = 0.16), the first reduction event (*E*_1/2_ = +0.25 V, *vs.* Ag/Ag^+^) resulted in a relatively low electron density with an average eru of 0.22 ± 0.02. This means that about one electron is produced per four to five repeat units of reduced unprotonated BBL-P. Further decrease of doping potential led to gradual increase of the electron density (Fig. 5a) showing an average doping level of 0.78 ± 0.07 eru upon the second reduction event; thus, suggesting that approximately three to four electrons are injected per four repeat units of reduced protonated BBL-P at the naphthalene imine sites. These findings indeed corroborate the spectroelectrochemistry results indicating that the electron density of the second redox process is about 3-fold higher than that of the first redox reaction. At an applied potential of −0.15 V (*vs.* Ag/Ag^+^) which corresponds to the third redox process, the doping level reached 1.33 ± 0.07 eru, which is equivalent to about five electrons are injected per four repeat units. These results indicate that multiply charged species must exist, which agrees with the optical absorption spectroscopy results.

It is important to note that the electron density as a function of pH is in excellent agreement with the observed CV and Pourbaix diagrams presented earlier. For example, a horizontal line representing a doping level of 0.75 eru was found to intersect the pH = 1.04 line at −0.086 V and the pH = 2.46 line at −0.27 V, both of which are the formal potentials of the second reduction event of BBL-P in the respective electrolyte solutions. Using the calculated electron density at three different pH values, we can also interpolate and generate a heat map to illustrate the number of electrons per repeat unit as a function of doping potentials and electrolyte pH as shown in Fig. 5b. The doping level of the parent BBL ladder polymer in neutral aqueous electrolyte has been previously shown to reach 0.75–1.00 eru at more negative doping potentials between −0.5 V and −0.9 V (*vs.* Ag/Ag^+^) depending on the polymer molecular weights and the electrolyte.^50,52^ In our case, by tuning the electrolyte's pH value, we can achieve comparable doping level (*i.e.* 0.75–1.00 eru) at significantly lower doping potentials suggesting that potential BBL-P OECT devices operating in acidic environment could have reduced threshold voltage and lower power consumption.

### Probing the nature of charge carriers in electrochemically n-doped BBL-P

**Fig. 6:**
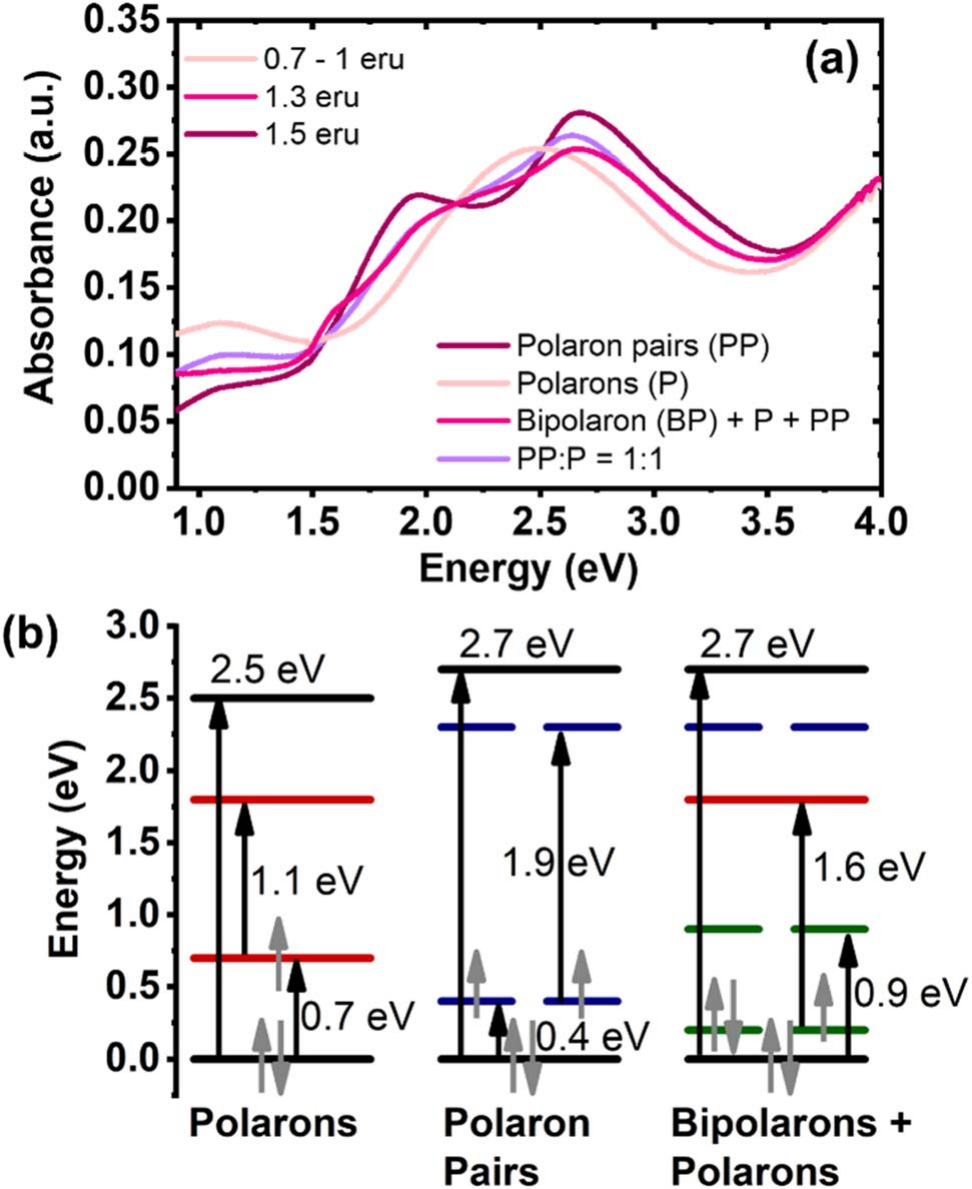
(a) Optical signatures of polarons (P), polaron pairs (PP), and singlet bipolarons (BP); (b) electronic band structure of different polaronic species including polaron, bipolaron, and polaron pairs.

The unique optical signatures of neutral, singly charged, and multiply charged species of BBL-P (Fig. 6a) from the above spectroelectrochemistry results enable us to track their formation upon electrochemical n-doping. In particular, Fig. 6a is the reconstruction of the spectroelectrochemistry results (Fig. 4b and c) in energy space (*E* = 1240/*λ*, where *E* is the energy (eV), and *λ* is the wavelength (nm)). The spectrum labeled 0.7–1.0 eru corresponded to the optical absorption spectrum collected with an applied potential of +0.05 V (*vs.* Ag/Ag^+^) in aqueous electrolyte of pH = 0.16 (Fig. 4b). The spectra labeled 1.3 eru and 1.5 eru corresponded respectively to the optical absorption spectra collected with an applied potential of −0.15 V (*vs.* Ag/Ag^+^) and −0.25 V (*vs.* Ag/Ag^+^) in electrolyte of pH = 0.16 (Fig. 4c). Based on the optical transition observed in Fig. 6a, we quantitatively constructed the evolution of the electronic band structure as a function of doping level (Fig. 6b).

Neutral BBL-P has a medium bandgap of 2.5 eV (Fig. S12a†) which is in good agreement with previously reported DFT results.^28^ As the polymer chains are reduced to an intermediate doping level (0.7–1.0 eru), which corresponds to the second redox event, a new absorption band arose centering at 1.1 eV (Fig. 6a and S12a†) which can be ascribed to the optical signature of polarons. Based on the observed optical transitions at 2.5 eV and 1.1 eV (Fig. S12a†), we proposed two possible electronic band diagrams of polarons (Fig. S12b†). In the first scenario, a bandgap (BG) transition corresponding to 2.5 eV is accompanied by a 1.1 eV transition from the valence band (VB) to the bonding state of the charged species (P1). This picture would mean that a finite gap of 0.3 eV (2.5–2 × 1.1 = 0.3) would remain (Fig. S12b†). In the second scenario, a BG transition at 2.5 eV is followed by a 1.1 eV transition from the bonding state to the anti-bonding state of the charged species (P2); thereby, leaving the P1 transition to be 0.7 eV (0.5 × (2.5 − 1.1) = 0.7). Most doped conjugated polymers are found to exhibit P2 transition with higher energy than P1 transition;^30,53,54^ thereby, we attributed the electronic band diagram presented in scenario 2 (Fig. S12b†) to that of polarons in doped BBL-P.

As the polymer is further reduced to a very high doping level (∼1.5 eru), the electronic band is widened from 2.5 eV to 2.7 eV (Fig. 6a and S13a†), which agrees with the formation of multiply charged species.^54^ In most doped conjugated polymers, multiply charged species can be categorized into either singlet bipolarons (BP) or triplet bipolarons which is also known as polaron pairs (PP). A recent study has suggested that strongly coupled polarons with lower energy P1 transition or red-shifted optical absorption could be more favorably formed in electrochemically doped poly(3-hexylthiophene) (P3HT) depending on the experimental conditions (*e.g.* potential bias, counterion size, counterion mobility, *etc.*).^30^ On the other hand, if such a transition has higher energy, it would be the optical signature of bipolarons agreeing with the traditional picture of doped conjugated polymers.^30,53–57^ When we assigned the 1.9 eV optical transition (Fig. S13a†) to the BP1 transition, the electronic band structure would be invalid as shown in scenario 1 of Fig. S13b† (1.9 × 2 > 2.7). This result suggests that bipolarons are unlikely to be the multiply charged species when BBL-P is doped to a high doping level of 1.5 eru. Therefore, the absorption peak at 1.9 eV (Fig. S13a†) is assigned to the transition from the VB to the anti-bonding state of the charged species; thus, implying that the gap between the VB and the bonding state of the charged species is 0.4 eV (0.5 × (2.7 − 1.9) = 0.4). This result means that the P1 transition seen in the multiply charged species has lower energy than that of polarons (Fig. S12b†); therefore, we assign strongly coupled polaron pairs (triplet bipolarons) rather than singlet bipolarons to be the charge carriers in heavily reduced BBL-P. This finding is in agreement with the reported DFT results for BBL polymer^58^ where triplet bipolarons are formed in highly doped samples.

As mentioned earlier in the spectroelectrochemistry results, the optical absorption spectra corresponding to the third redox event of BBL-P (1.3 eru) were severely distorted, suggesting the coexistence of different polaronic species. We thus performed linear superpositions of the optical signatures of polaron (P) and polaron pairs (PP) to quantify the distribution of these polaronic species in the reduced BBL-P films. Despite varying PP : P compositions, the superimposed spectra could only match the main electronic excitations in the visible (4–1.7 eV) and near-IR (0.9–1.5 eV) regions and failed to capture the electronic transition at 1.6 eV (Fig. S8b†). This result suggests that there must be some electronic interactions between polarons and polaron pairs to give rise to a new charged species with an electronic transition of 1.6 eV (Fig. S14a†). Indeed, two isosbestic points at 1.5 eV and 1.7 eV (Fig. S14a†) correspond to the transformation of polaron pairs and polarons into a new charged species. In other words, the third redox process of BBL-P should consist of a ternary mixture of polaron, polaron pairs, and a new multiply charged species.

We propose that this new multiply charged species are singlet bipolarons arising from the hybridization between the bonding states of polarons and polaron pairs (Fig. S14b†). The optimal energy level distribution of polarons and polaron pairs enables the formation of two new mid-gap levels at *E*_1_ = 0.2 eV and *E*_2_ = 0.9 eV (Fig. S14b†) whereby *E*_1_ + *E*_2_ = *P*_1, polaron_ + *P*_1, polaron pairs_ = 1.1 eV by virtue of conservation of energy. Thus, the 1.6 eV transition can be assigned to the transition from the new hybridized *E*_1_ state to the non-bonding state of polarons (Fig. 6b and S14b†). These findings provide the first experimental evidence of the coexistence of polarons, polaron pairs, and bipolarons in an n-type π-conjugated polymer and clearly mapped out the transition between each polaronic species in energy space. We note that previously reported DFT calculations have predicted the coexistence of polarons and bipolarons on the same polymer chain of BBL;^58^ however, the calculation did not provide a mechanism as to how polarons can turn into bipolarons.

In summary, we have experimentally provided a complete picture of the formation and transition of different polaronic species in an n-doped π-conjugated polymer. At a low to intermediate doping level of 0.1–1.0 eru, polarons are the main charge carrier, and they show two mid-gap transitions of 1.1 eV and 0.7 eV, which corresponds to the electronic transition from the valence band to the polaron nonbonding state and the polaron bonding state, respectively. At a very high doping level of 1.5 eru, strongly coupled polaron pairs, rather than bipolarons, are the dominant charge carriers, and they show a wider bandgap accompanied by two mid-gap transitions of 0.4 eV and 1.9 eV. At an intermediate-high doping level of ∼1.3 eru, polarons and polaron pairs coexist in equal amount, and the hybridization between their bonding states gives rise to singlet bipolarons; thus, a ternary combinations of polarons, polaron pairs, and bipolarons are in equilibrium.

### *In operando* Raman spectroscopy of BBL-P films

**Fig. 7:**
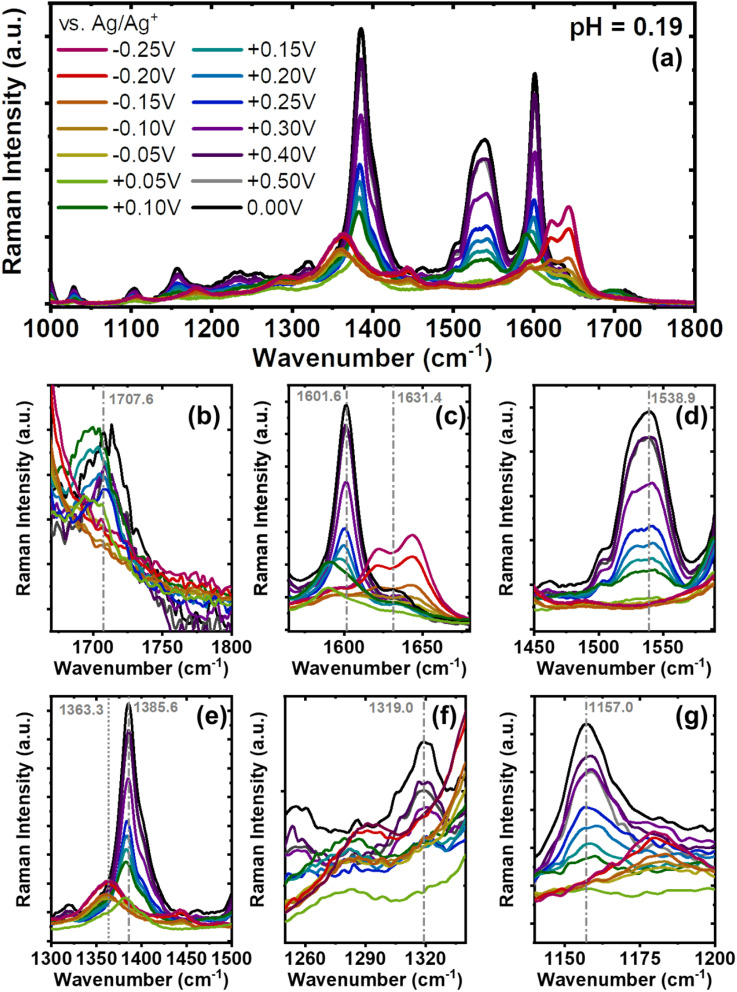
(a) *In operando* Raman spectra of BBL-P films with 532 nm excitation in the range of 1000 –1800 cm^−1^ region. (b–g) Zoomed in Raman spectra of BBL-P films in the regions of (b) 1125–1220 cm^−1^, (c) 1250–1350 cm^−1^, (d) 1300–1500 cm^−1^, (e) 1450–1590 cm^−1^, (f) 1565–1680 cm^−1^, and (g) 1670–1800 cm^−1^. Raman measurements were collected from BBL-P films coated on gold covered glass substrates as the working electrode with 0.1 M KCl_(aq)_ (pH = 0.19) as the electrolyte, Pt mesh as the counter electrode, Ag/AgCl pellet as reference electrode positioned away from the illuminated area.

We then used *in operando* Raman spectroscopy to probe the molecular structure and electronic structure evolution of BBL-P upon electrochemical doping. We will similarly focus our discussion on results obtained in solution of pH < 1 as all three redox states of BBL-P coexist in equilibrium under this condition. The Raman spectra of BBL-P in highly acidic electrolyte (pH = 0.19) are shown in Fig. 7, and the close-up spectra of relevant vibrational modes are shown in Fig. 7b–g. The Raman spectra of BBL-P in electrolyte of higher pH values (pH ∼2 and 3) are shown in Fig. S15.† The Raman intensity decreased over the whole measurement ranges indicating the formation of polaronic species upon electrochemical reduction.^52,59^ The most intense Raman vibrational modes of undoped BBL-P are located at *w*_1_ = 1713 cm^−1^, *w*_2_ = 1630 cm^−1^, *w*_3_ = 1601 cm^−1^, *w*_4_ = 1538 cm^−1^, *w*_5_ = 1385 cm^−1^, *w*_6_ = 1319 cm^−1^, and *w*_7_ = 1157 cm^−1^. The *w*_1_ Raman mode can be assigned to the symmetric C=O vibrations which gradually decreased and was completely bleached at *E* < +0.05 V (*vs.* Ag/Ag^+^) (Fig. 7b). The disappearance of the *w*_1_ vibrational modes implies a very high doping level beyond 1.0 eru was obtained at more negative potential,^52^ which corroborates well with our coulometry results.

The *w*_2_ Raman mode (1630 cm^−1^) can be assigned to the C=N stretch arising from both the naphthalene ring and the phenazine unit^60^ whereas the *w*_3_ mode (1601 cm^−1^) originates from the C=C/C–C vibrations. Upon electrochemical doping, the *w*_3_ peak intensity decreased and gradually shifted to lower frequency (Fig. 7c and S16a†). In particular, the *w*_3_ Raman band was found at 1589 cm^−1^ (12 cm^−1^ shift) upon the second redox reaction (*E* = +0.05 V, *vs.* Ag/Ag^+^). The substantial frequency decrease suggests that the polarons that are formed cause the structure to adopt a more quinoidal character.^58,61,62^ As the polymer chains are further reduced (*E* = −0.15 to −0.20 V, *vs.* Ag/Ag^+^), the *w*_3_ vibrational mode increased in intensity and shifted back to higher frequency centering at 1620 cm^−1^ (Fig. 7c and S16a†). This result implies that the strongly coupled polaron pairs and bipolarons that are formed upon the third redox event results in a pro-benzoidal structure. As a result, these multiply charged species would be much less delocalized compared to polarons.

Moreover, the *w*_2_ Raman band (1630 cm^−1^) was found to progressively shift to higher wavenumber as the applied potential was swept from positive to negative (Fig. S16b†). In particular, this vibrational mode centered at 1637 cm^−1^ for *E* = +0.05 V (*vs.* Ag/Ag^+^) and at 1663–1664 cm^−1^ for *E* = −0.15 to −0.20 V (*vs.* Ag/Ag^+^). The significant red-shift is indicative of the protonation of the imine bond to yield –C=N^+^– on both the phenazine rings and the naphthalene rings.^63^ The higher Raman intensity and more red-shifted Raman band seen at −0.15 to −0.20 V compared to that at +0.05 V suggests that the concentration of –C=N^+^– is higher in this reduced form of BBL-P. We note that at low doping level (*E* = +0.25 V, *vs.* Ag/Ag^+^), the –C=N– vibrational mode exhibited marginal shift which means that very few number of imine sites are protonated at this potential.

The *w*_4_ mode (1538 cm^−1^) corresponds to the aromatic C–C and C–N skeletal vibrations. The slight intensity decrease seen at *E* = +0.25 V (Fig. 7d) suggests that there are marginal conformational changes in the polymer backbone, which makes sense given the low doping level (∼0.2 eru). However, the *w*_4_ Raman band was completely bleached at more negative potentials (*E* < +0.05 V) suggesting significant structural changes upon the formation of charged species.^59^ This finding indeed corroborates the high doping level (>1.0 eru) explained by coulometry earlier.

The *w*_5_ mode (1385 cm^−1^) can be assigned to the –C–N– single bond stretching.^28^ As the doping level increased, the Raman intensity was found to gradually decrease, and the peak position shifted to lower wavenumber (Fig. 7e and S16c†). In particular, the peak shifting can be divided into three regions where the first region (*E* > 0.3 V, *vs.* Ag/Ag^+^) showed minimal change in frequency, the second region (0 V < *E* < 0.3 V, *vs.* Ag/Ag^+^) featured a 4 cm^−1^ shift, and the third region (*E* < 0 V, *vs.* Ag/Ag^+^) had a substantial shift of 23 cm^−1^ (Fig. S16c†). The significant peak shift of this –C–N– vibrational mode seen in the second and third region is indicative of formation of charged species or –C–Ṅ^+^– as previously reported for polyaniline and its derivatives.^61,64,65^ Moreover, the substantial peak shift corroborates the formation of strongly coupled polaron pairs and bipolarons upon the third redox reaction, which can be explained by an increase of conducting (charged) environment around the –C–N– stretch,^66^ whereas polarons are the main charge carrier in the second redox reaction.

The *w*_6_ Raman mode (1319 cm^−1^) in undoped BBL-P can be assigned to the –C–N– stretching of the phenazine ring. This vibrational mode was shifted to much lower energy (1285 cm^−1^) (Fig. 7f), which can be ascribed to the formation of polaronic species. Lastly, the *w*_7_ Raman mode (1157 cm^−1^), which corresponds to in-plane C–H bending, was substantially upshifted to 1179 cm^−1^ upon the third reduction reaction (Fig. 7g). This large increase in vibrational energy of this mode can be explained by the –C–H– vibrational of the radical cation segments.

In summary, our *in operando* Raman spectroscopy results provide important insights on the molecular and electronic structures of BBL-P upon electrochemical doping, all of which are found to be in excellent agreement with the spectroelectrochemistry and coulometry analyses. The first reduction reaction of BBL-P results in minimal structural changes mainly due to the low and non-uniform distribution of polarons across the polymer backbone (1 electron per 4–5 repeat units). The second reduction reaction of BBL-P leads to the formation of polarons and induces significant changes in the molecular structures, including more quinoidal characteristics and an intermediate concentration of protonated imine sites. The third reduction reaction of BBL-P, involving the formation of polarons, polaron pairs, and bipolarons, results in greater structural changes, including more benzoidal characteristics and a high concentration of protonated imine sites.

### Proposed schemes for BBL-P electrochemical reactions

Taking all the above data together, we now provide a comprehensive scheme to explain the multiple electrochemical reduction reactions of BBL-P in acidic aqueous electrolytes.

**Scheme 2:**
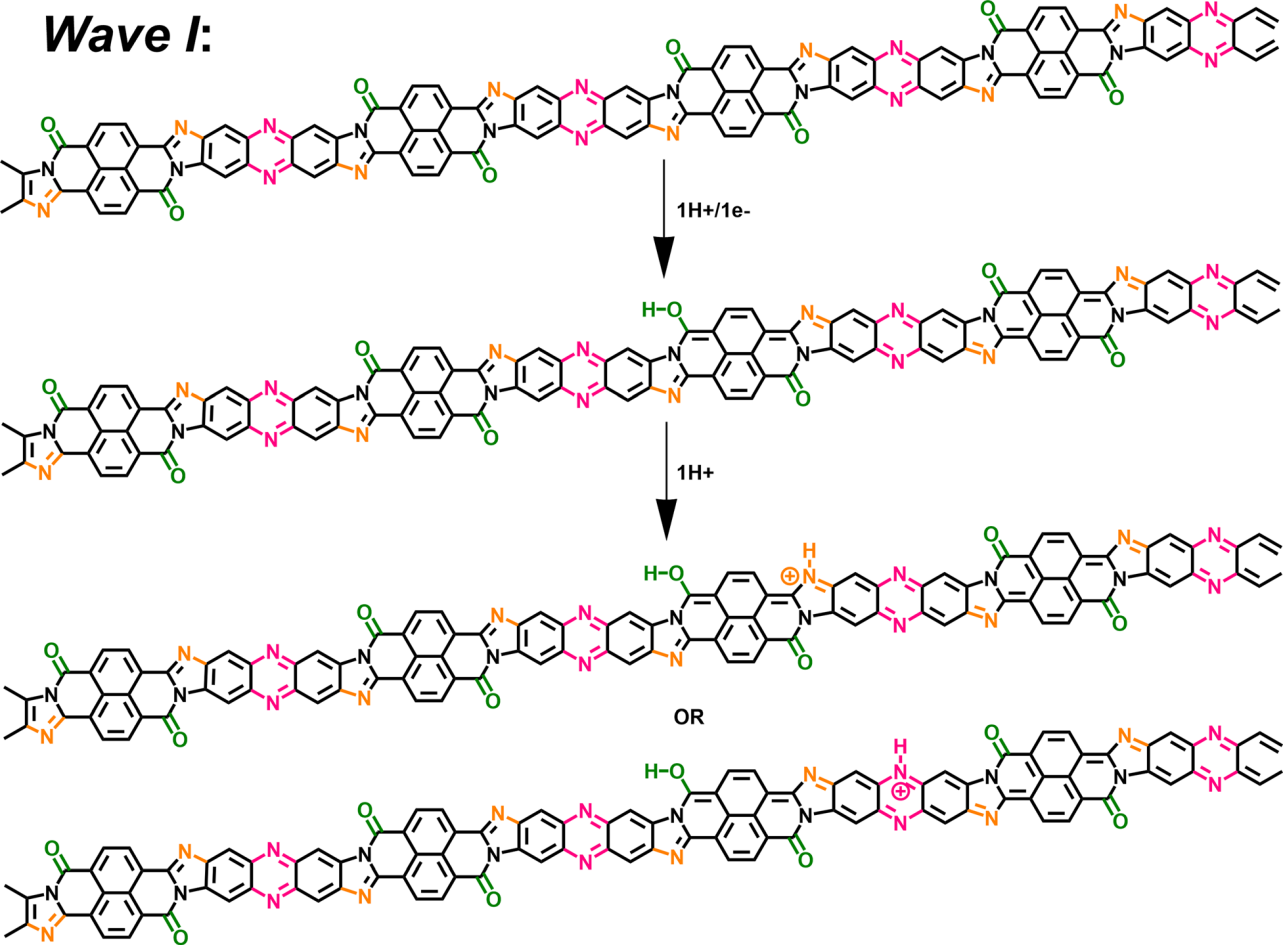
A two-proton/one-electron electrochemical doping reaction (wave I) of BBL-P consisting of 1H^+^/1e^−^ at the carbonyl oxygen sites followed by 1H^+^ either at the naphthalene imine or the phenazine imine sites.

At pH > 3, the BBL-P film remains unprotonated and facilely undergoes a redox reaction to form the first reduced form (Scheme 2). At this stage, the doping level is rather low, featuring about 1 electron per 4–5 repeat units as suggested by spectroelectrochemistry and coulometry results. As a result, minimal changes in the molecular structure are observed as evidenced by the *in operando* Raman spectroscopy analysis.

**Scheme 3:**
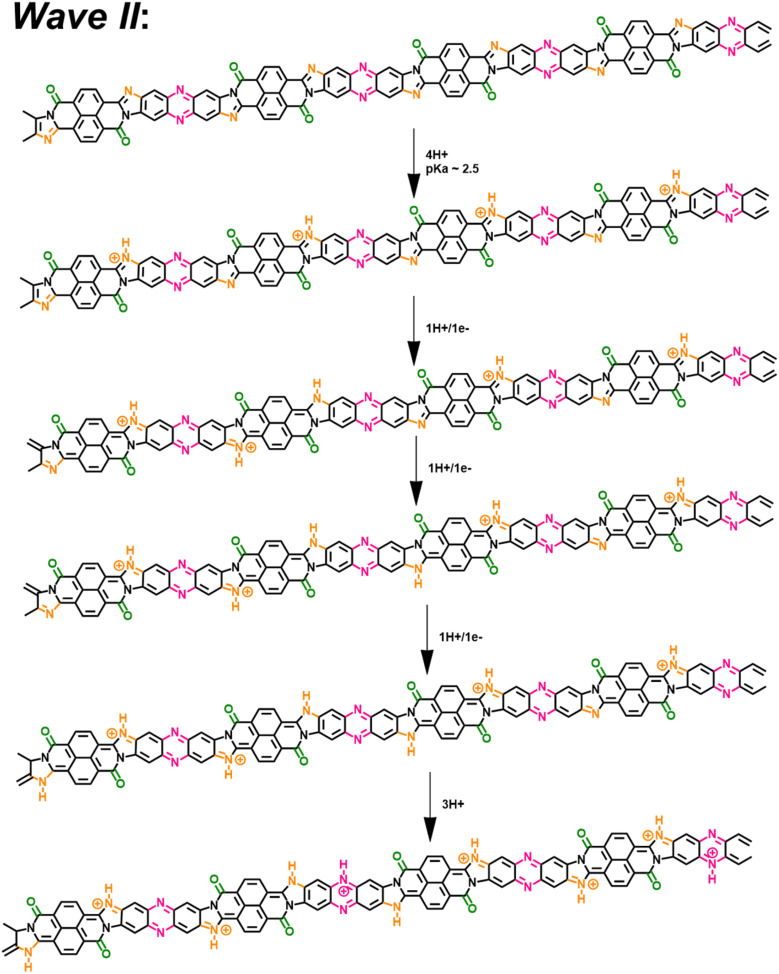
A two-proton/one-electron electrochemical doping reaction (wave II) of BBL-P comprising of 1H^+^/1e^−^ transferred at each naphthalene imine site followed by 1H^+^ at the phenazine imine sites.

At 1 < pH < 3, the BBL-P is partially protonated, specifically at the naphthalene imine bond and then undergoes a redox reaction at a more negative potential to form the second reduced form. The density of polarons in this structure is shown to be around 3–4 electrons per 4 repeat units *via* spectroelectrochemistry and coulometry. *In operando* Raman spectroscopy reveals that this reduced form adopts a more quinoidal character than the undoped unprotonated BBL-P. The molecular structure of this reduced form is thus shown in Scheme 3.

**Scheme 4:**
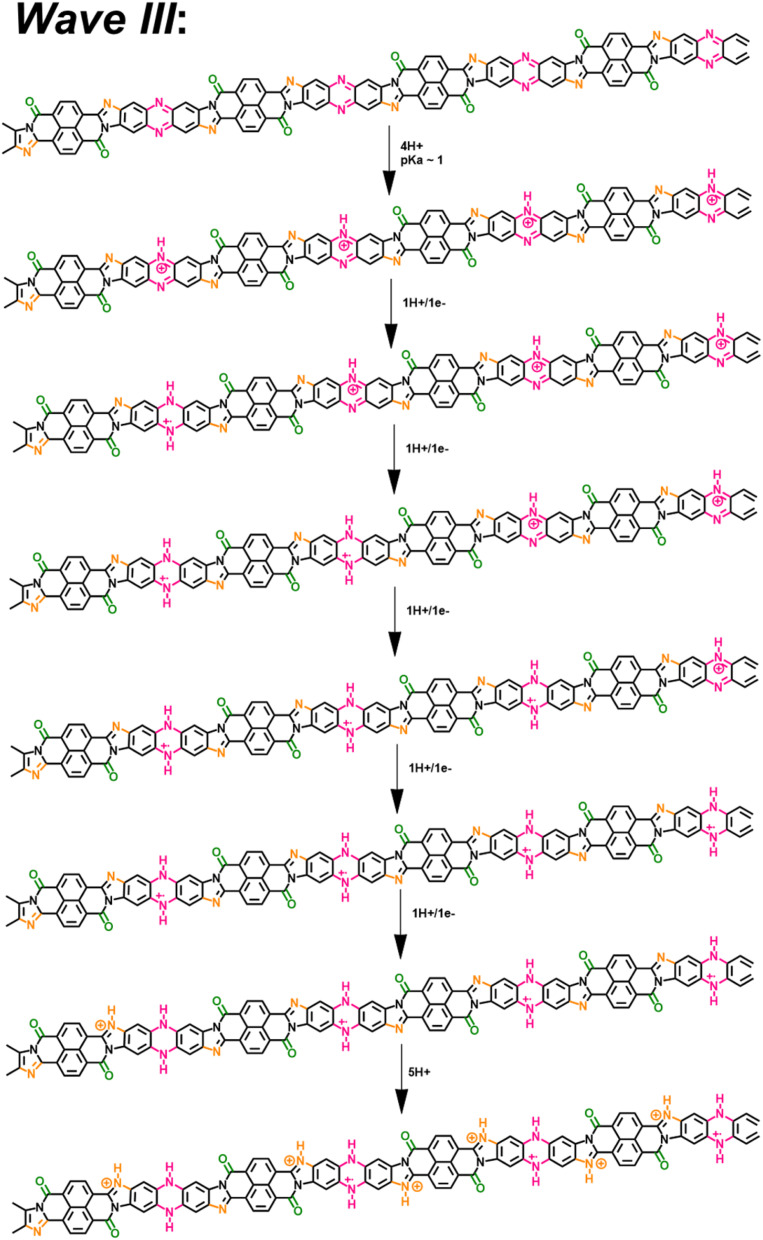
A two-proton/one-electron electrochemical doping reaction (wave III) of BBL-P consisting of a 1H^+^/1e^−^ transferred at each phenazine imine sites accompanied by 1H^+^ at the naphthalene imine sites.

At pH < 1, the imine bonds of the phenazine rings are protonated, and the protonated polymer undergoes a redox reaction to form the third reduced form at significantly more negative potential. Multiply charged BBL-P with a high doping level of approximately 5 electrons per 4 repeat units are found *via* spectroelectrochemistry and coulometry. The polymer structure of this reduced state preferentially adopts a more benzoidal character that features a large concentration of –C=N^+^– sites on both the phenazine rings and the naphthalene rings. The molecular structure of this reduced state of BBL-P is thus illustrated in Scheme 4.

It is important to reiterate that all three final products are formed as a result of two-proton per one-electron transferred redox reactions, which is to be contrasted with commonly seen one-proton/one-electron PCET. The extra proton insertion to the polymer backbone implies that all reduced products of BBL-P should have higher redox capacity and volumetric capacitance than the parent BBL.^67,68^ Our present results have thus established important fundamental frameworks regarding the electrochemical reactivity and resulting optoelectronic and structural evolution of BBL-P, all of which could be broadly applicable to device applications.

### Redox capacity of BBL-P films as a function of pH and applied potential

We have also explored the charge storage capability of BBL-P in acidic environment which have implications for applications in organic electrochemical transistors (OECTs), all-organic proton batteries, and proton supercapacitors. The redox capacity (*ρ*) is defined as the change in volumetric charge density per infinitesimal change of equilibrium potential relative to a reference electrode:^67,68^
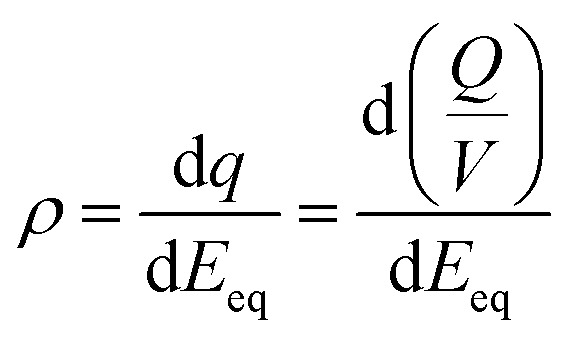
where *ρ* is the redox capacity (F cm^−3^), *Q* is the charge stored in the electrode (C), *V* is the volume of the electrode (cm^3^), and *E*_eq_ is the equilibrium potential.

**Fig. 8:**
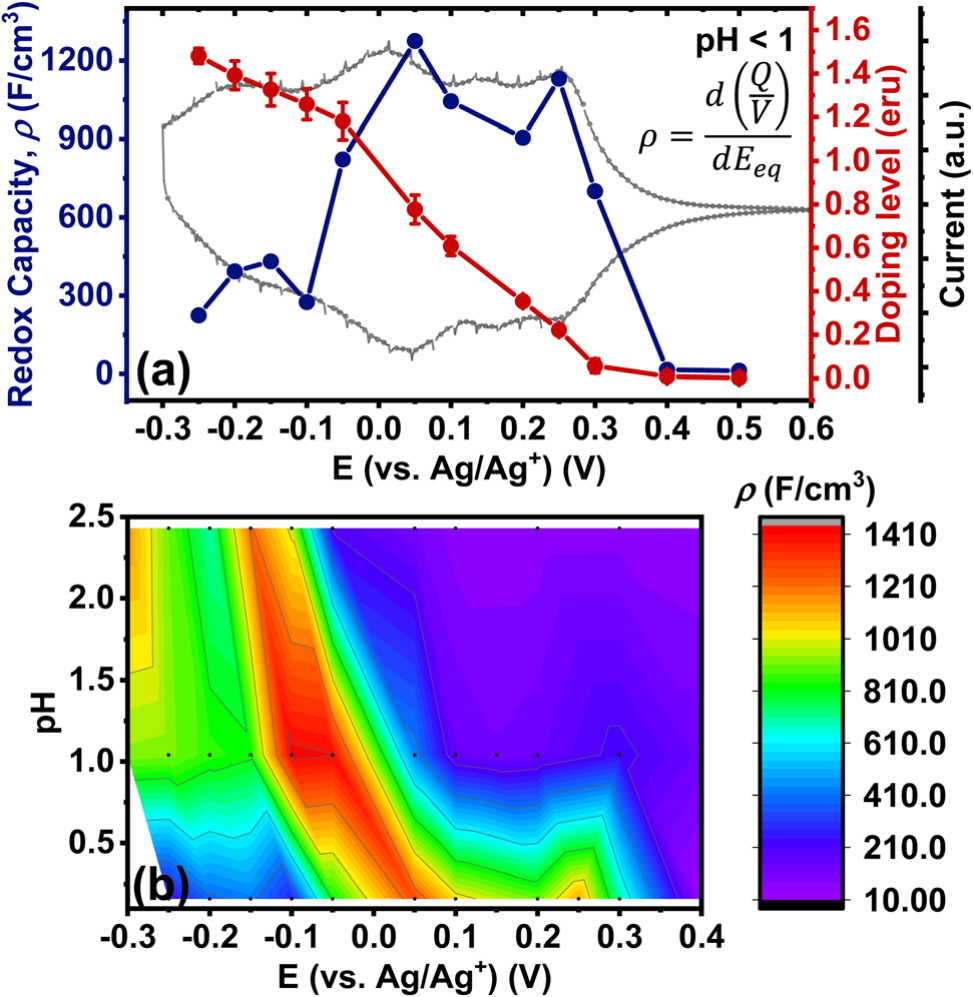
(a) Evolution of redox capacity (*ρ*) and doping level (eru) of BBL-P thin films as a function of doping potential in electrolyte of pH < 1; (b) dependence of redox capacity of BBL-P thin films as a function of electrolyte pH and doping potentials.

Using the total charge obtained from coulometry analysis, we computed the redox capacity of BBL-P with respect to the doping potential in electrolyte of pH < 1 as shown in Fig. 8a, and the heatmap showing the evolution of redox capacity as a function of both doping potential and electrolyte pH is illustrated in Fig. 8b. The redox capacity of BBL-P in highly acidic electrolyte exhibited three different peaks at around +0.25 V, +0.05 V, and −0.15 V (*vs.* Ag/Ag^+^) which coincided with the formal potential of the first, second, and third redox event, respectively. This result makes sense since electrons are injected into the polymer films at these potentials. Moreover, the maximum *ρ* value of BBL-P in acidic electrolyte (*ρ* = 1272 F cm^−3^) is significantly higher than that of the parent BBL in neutral aqueous environment (540–1000 F cm^−3^)^50,69^ and neutral non-aqueous environment (∼150 F cm^−3^).^70^ We note that the molecular weight of BBL-P used in this study is considered to be relatively low with an intrinsic viscosity around 1.1 dL g^−1^; thus, further enhanced redox capacity can be expected if higher molecular weight sample is synthesized.^50^ Our present results demonstrate that the unique molecular structure of BBL-P, which features multiredox sites with varying p*K*_a_ values, enables a remarkably high redox capacity upon protonation and reduction.

## Conclusions

We have investigated the proton-coupled electron transfer (PCET) process involved in electrochemical n-doping of a novel multiredox π-conjugated polymer, phenazine-substituted poly(benzimidazobenzophenanthroline) (BBL-P), in aqueous electrolyte. From the measured Pourbaix diagram of each of the three redox processes in BBL-P, we have discovered that the PCET response is super-Nernstian, exhibiting two protons per one electron transferred. Although super-Nernstian PCET has previously been observed in some hydrated transition metal oxides^9–12^ and metalloproteins,^4,13,14^ our present results represent the first observation of super-Nernstian PCET behavior in a purely organic material/polymer.

We have also combined pH-dependent spectroelectrochemistry, *in operando* Raman spectroscopy, and coulometry to probe the nature of charge carriers in the electrochemically n-doped BBL-P. We found that polarons, delocalized over a pro-quinoidal polymer backbone, are the charge carriers at low to intermediate doping levels (0.1–1.0 eru). At a higher doping level (1.3 eru), a ternary mixture of polarons, polaron pairs, and bipolarons were found to co-exist in equilibrium. At the highest doping level (1.5 eru), polaron pairs were found to be the dominant charge carriers.

Finally, our pH-dependent coulometry analysis of BBL-P showed that it has a very high redox capacity (>1200 F cm^−3^) in aqueous acidic (pH < 1) electrolyte, which suggests good potential for applications in energy storage and other electrochemical devices.

## Data availability

The data that support the findings of this study are available within the paper and its ESI.† Additional data may be requested from the authors.

## Author contributions

D. K. T. conceived the idea, investigated, conducted the experiments (CV, spectroelectrochemistry, coulometry, *in operando* Raman spectroscopy), and performed formal analysis and visualization of the experimental data and DFT results. S. M. W. synthesized the polymer and performed Density Functional Theory (DFT) calculations. E. M. K. S. assisted with the synthesis of the polymer. S. A. J. supervised all aspects of the study. The manuscript was written through contributions of all authors. All authors have given approval to the final version of the manuscript.

## Conflicts of interest

The authors declare no competing financial interest.

## Supplementary Material

SC-015-D4SC00785A-s001
