# Supplementary material for: Observation of super-Nernstian proton-coupled electron transfer and elucidation of nature of charge carriers in a multiredox conjugated polymer
Source: Chem Sci. 2024 Apr 22;15(20):7623–42. doi: 10.1039/d4sc00785a (PMC11110174; doi:10.1039/d4sc00785a)
Supplement: SC-015-D4SC00785A-s001 [file SC-015-D4SC00785A-s001.pdf]

Electronic Supplementary Material (ESI) for Chemical Science.
This journal is © The Royal Society of Chemistry 2024

Supporting Information

Observation of Super-Nernstian Proton-Coupled Electron Transfer and Elucidation of Nature of Charge Carriers in a Multiredox Conjugated Polymer

Duyen K. Tran,[a] Sarah M. West,[b] Elizabeth M. K. Speck,[b] and Samson A. Jenekhe[*, a, b]

[a]Department of Chemical Engineering, University of Washington, Seattle, Washington 98195, United States

[b]Department of Chemistry, University of Washington, Seattle, Washington 98195, United States

TABLE OF CONTENTS

SUPPORTING FIGURES

Figure S1. pH-dependent cyclic voltammograms pf BBL-P thin films collected in 0.1 M $KCl_{(aq)}$ supporting electrolyte: (a) pH = 8.12, (b) pH = 6.99, (c) pH = 6.50, and (d) pH = 4.51. .... S3

Figure S2. Method to extract the formal potential ($E_{1/2}$) of each pair of redox waves.............................. S3

Figure S3. pH-dependent cyclic voltammograms of BBL thin films collected in 0.1M $KCl_{(aq)}$ supporting electrolyte with a scan rate of 25 mV/s using a 3-electrode configuration with ITO/BBL as the working electrode, Pt mesh as the counter electrode, and Ag/AgCl as the reference electrode. S4

Figure S4. Representative Pourbaix diagram of BBL thin films in $0.1M_{(aq)}$ KCl electrolyte at a scan rate of 25 mV/s... S4

Figure S5. DFT calculations of the optimized ground-state molecular geometry of different redox states of BBL-P dimers: (a) neutral BBL-P corresponding to structure 1 to Scheme 2, (b) reduced unprotonated BBL-P, (c) reduced protonated BBL-P at the naphthalene imine sites, and (d) reduced protonated BBL-P at the phenazine imine sites. Gas-phase molecule DFT calculations were performed using the Gaussian 16 suit of programs.[1] Calculations were performed at the ωB97XD/6-31G(d,p) level of theory................... S4

Figure S6. Gibbs free energy change (ΔG) for each acid-base coupled redox reaction of BBL-P. S5

Figure S7. Relationship between log(i) and log (ν) for different redox event at various electrolyte pH. .. S5

Figure S8. (a) Thin film optical absorption spectra of BBL-P in 0.1M KCl aqueous electrolyte solution of varying pH 2.46, 1.04, and 0.16; (b) Optical absorption spectra of BBL-P thin films at different varying composition of polaron pairs (PP) and polarons (P). S6

Figure S9. (a) Cyclic voltammogram of BBL-P thin films in aqueous KCl electrolyte of pH 2.51 collected at a scan rate of 25 mV/s; (b – c) UV-Vis-NIR optical absorption spectra under different potentials of BBL-P thin films in electrolyte of pH ~ 2.5; (d – e) Differential UV-Vis-NIR optical absorption spectra under different potentials of BBL-P thin films. The baseline spectrum was taken at +0.50V to de-dope the films prior to each doping step.. S6

Figure S10. (a) Cyclic voltammogram of BBL-P thin films in aqueous KCl electrolyte of pH 1.07 collected at a scan rate of 25 mV/s; (b – c) UV-Vis-NIR optical absorption spectra under different potentials of BBL-P thin films in electrolyte of pH = 1.04; (d – e) Differential UV-Vis-NIR optical absorption spectra under different potentials of BBL-P thin films. The baseline spectrum was taken at +0.50V to de-dope the films prior to each doping step... S7

Figure S11. Dependence of differential absorbance of BBL-P in electrolyte of pH < 1 on doping potential at selected wavelength: (a) 1115 nm and (b) 461 nm and 640 nm..S7

Figure S12. (a) Optical signatures of neutral BBL-P and polarons in doped BBL-P; (b) Possible electronic band structure of polarons in doped BBL-P. S8

Figure S13. (a) Optical signatures of multiply charged species in doped BBL-P; (b) Possible electronic band structure of multiply charged species, bipolaron (BP) or polaron pairs (PP), in doped BBL-P.........S9

Figure S14. (a) Optical signatures of a mixture of singly charged and multiply charged species in doped BBL-P; (b) Hybridization between the bonding state of polarons and polaron pairs to yield bipolarons in doped BBL-P films. S10

Figure S15. *In-operando* Raman spectra of BBL-P films collected in 0.1M $KCl_{(aq)}$ electrolyte of varying pH value: (a) pH = 1.99, and (b) pH = 2.80. Raman measurements were collected using 532 nm excitation with BBL-P films coated on gold covered glass substrates as the working electrode, Pt mesh as the counter electrode, Ag/AgCl pellet as reference electrode positioned away from the illuminated area. ...S11

Figure S16. (a) Evolution of Raman frequency and Raman intensity associated with the C=C/C-C breathing of the naphthalene ring as a function of doping potential; (b) Evolution of Raman frequency and Raman intensity associated with the C=N vibrational mode as a function of doping potential; (c) Raman signatures of singly charged species (polaron) and multiply charged species (coupled polaron pairs)....S12

SUPPORTING TABLES

Table S1. Quantification of Proton-Coupled Electron Transfer Processes of BBL Thin Films...............S13

Table S2. Summary of reduction potential of some redox active molecules. S13

Table S3. Density Functional Theory (DFT) Computed Atomic Charge Distribution of BBL-P Dimers in its Neutral State (Structure 1), Reduced at Carbonyl Oxygen State (Structure 1a), and Protonated Reduced State (Structure 2). S14

Table S4. Density Functional Theory (DFT) Computed Atomic Charge Distribution of BBL-P Dimers in its Neutral State (Structure 1), Protonated at Naphthalene Imine (Structure 3), Reduced Protonated at Naphthalene Imine (Structure 4a), and Reduced Double Protonated at Naphthalene Imine and Phenazine Imine (Structure 4b). S15

References...S15

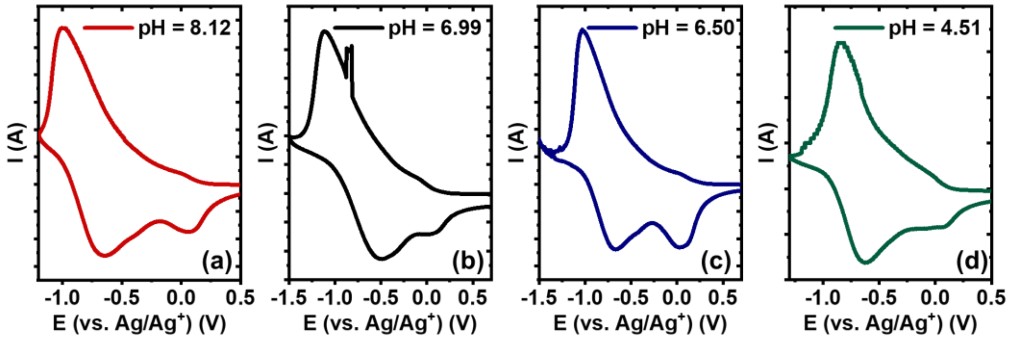

Figure S1. pH-dependent cyclic voltammograms pf BBL-P thin films collected in 0.1 M $KCl_{(aq)}$ supporting electrolyte: (a) pH = 8.12, (b) pH = 6.99, (c) pH = 6.50, and (d) pH = 4.51.

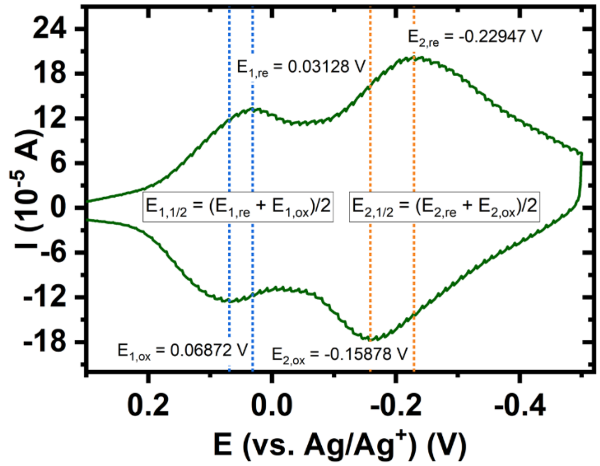

Figure S2. Method to extract the formal potential ($E_{1/2}$) of each pair of redox waves.

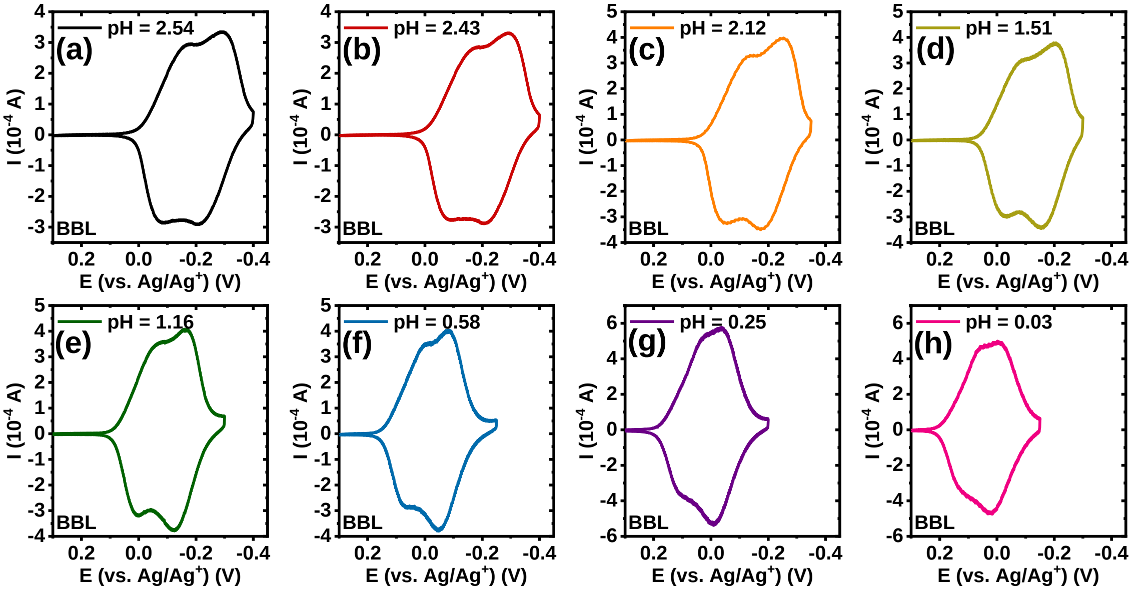

Figure S3. pH-dependent cyclic voltammograms of BBL thin films collected in 0.1M $KCl_{(aq)}$ supporting electrolyte with a scan rate of 25 mV/s using a 3-electrode configuration with ITO/BBL as the working electrode, Pt mesh as the counter electrode, and Ag/AgCl as the reference electrode.

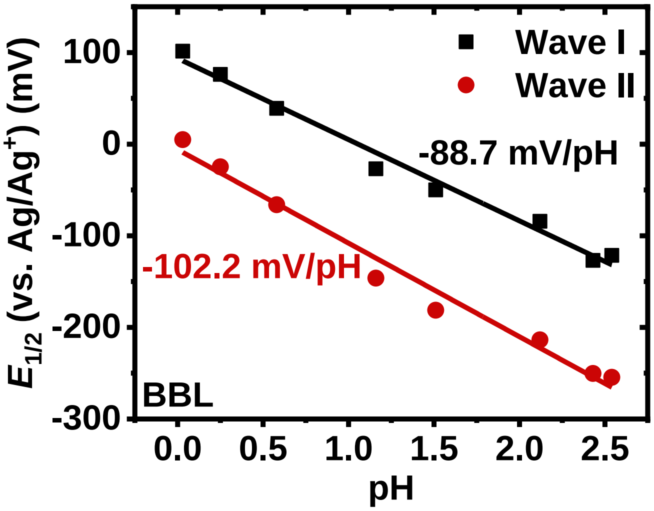

Figure S4. Representative Pourbaix diagram of BBL thin films in $0.1M_{(aq)}$ KCl electrolyte at a scan rate of 25 mV/s.

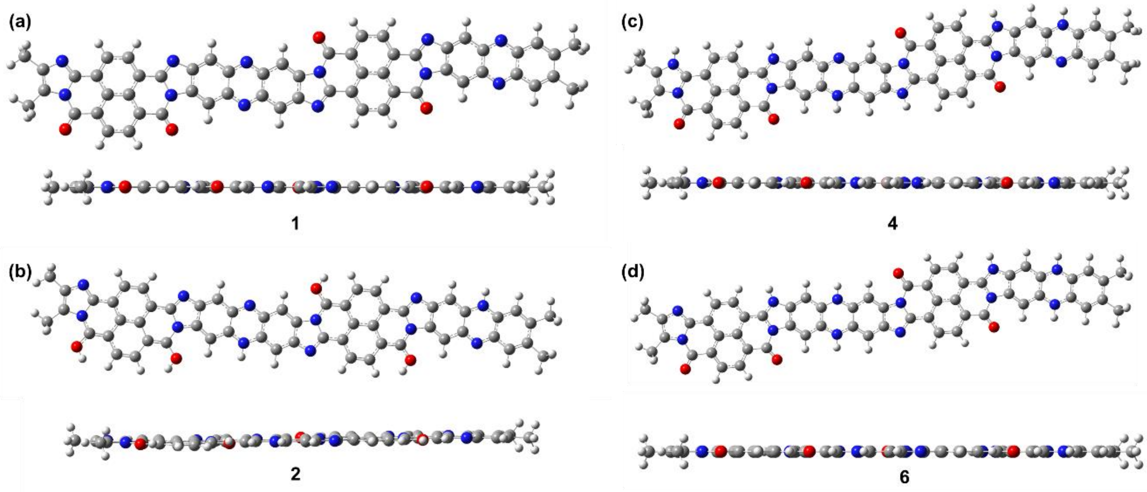

Figure S5. DFT calculations of the optimized ground-state molecular geometry of different redox states of BBL-P dimers: (a) neutral BBL-P corresponding to structure 1 to Scheme 2, (b) reduced unprotonated BBL-P, (c) reduced protonated BBL-P at the naphthalene imine sites, and (d) reduced protonated BBL-P at the phenazine imine sites. Gas-phase molecule DFT calculations were performed using the Gaussian 16 suit of programs.[1] Calculations were performed at the ωB97XD/6-31G(d,p) level of theory.

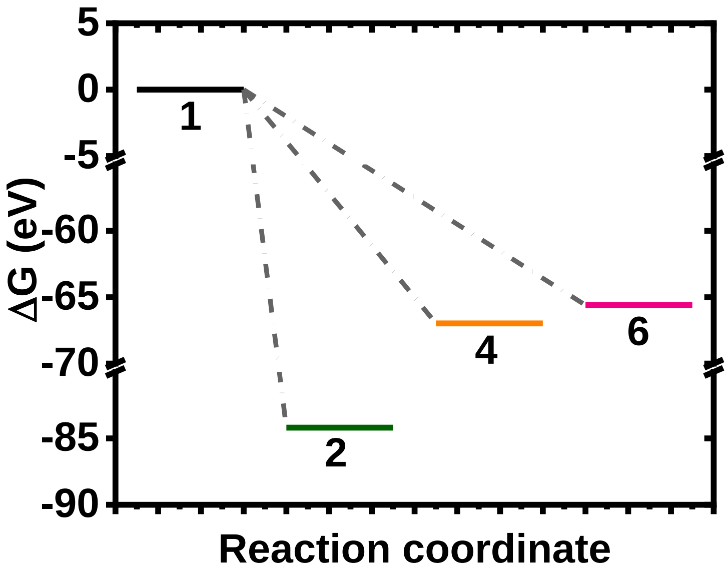

Figure S6. Gibbs free energy change (Δ*G*) for each acid-base coupled redox reaction of BBL-P.

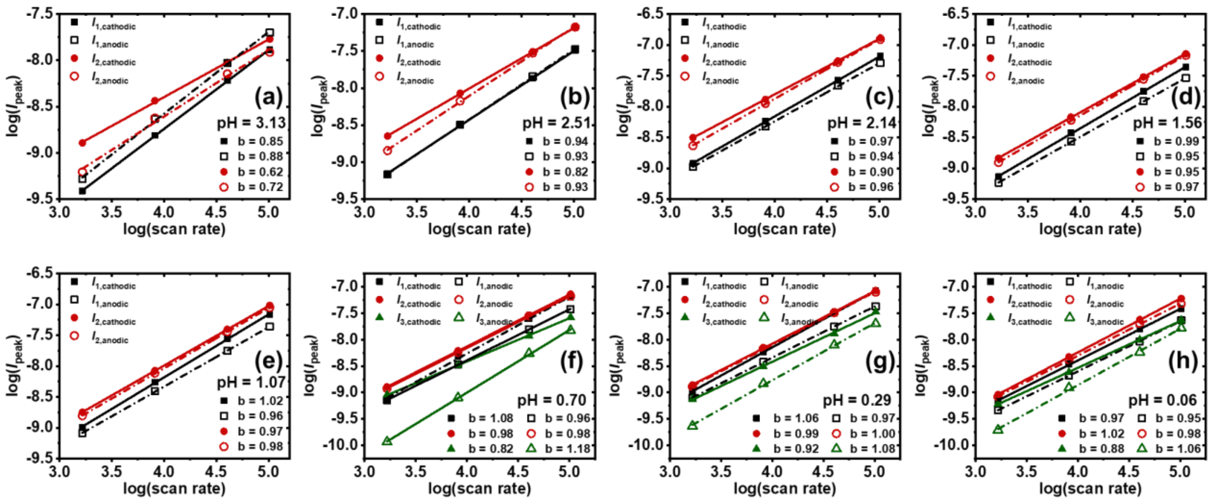

Figure S7. Relationship between log(*i*) and log (ν) for different redox event at various electrolyte pH.

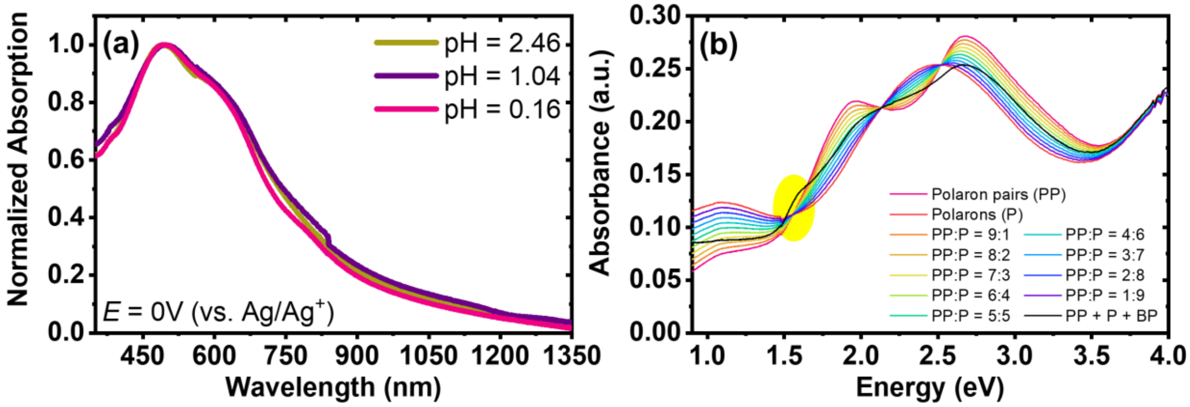

Figure S8. (a) Thin film optical absorption spectra of BBL-P in 0.1M KCl aqueous electrolyte solution of varying pH 2.46, 1.04, and 0.16; (b) Optical absorption spectra of BBL-P thin films at different varying composition of polaron pairs (PP) and polarons (P).

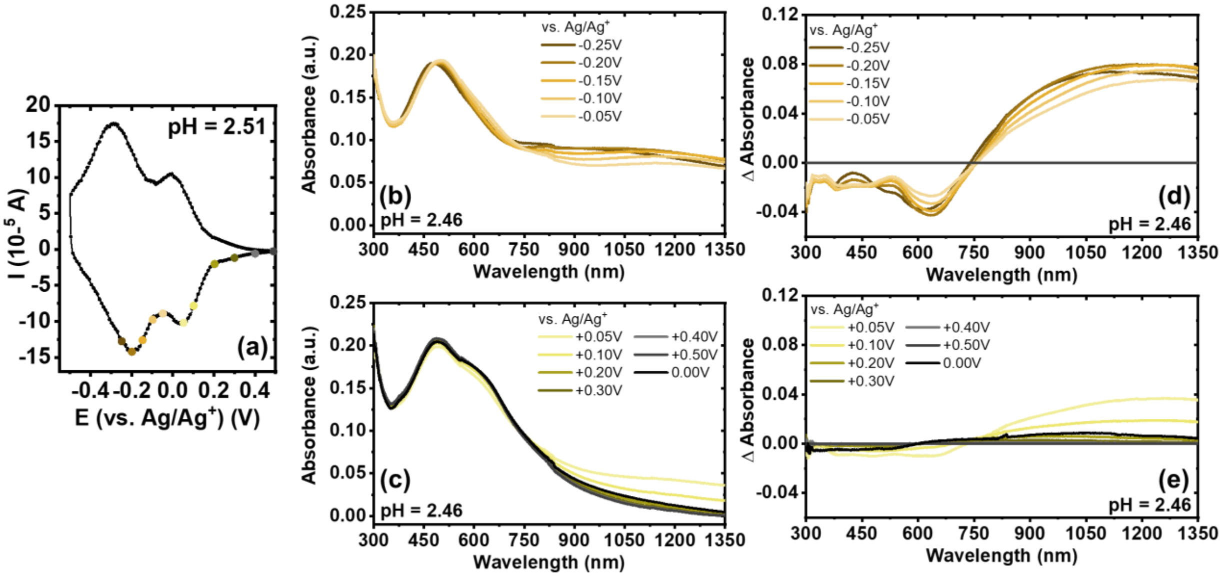

Figure S9. (a) Cyclic voltammogram of BBL-P thin films in aqueous KCl electrolyte of pH 2.51 collected at a scan rate of 25 mV/s; (b – c) UV-Vis-NIR optical absorption spectra under different potentials of BBL-P thin films in electrolyte of pH ~ 2.5; (d – e) Differential UV-Vis-NIR optical absorption spectra under different potentials of BBL-P thin films. The baseline spectrum was taken at +0.50V to de-dope the films prior to each doping step.

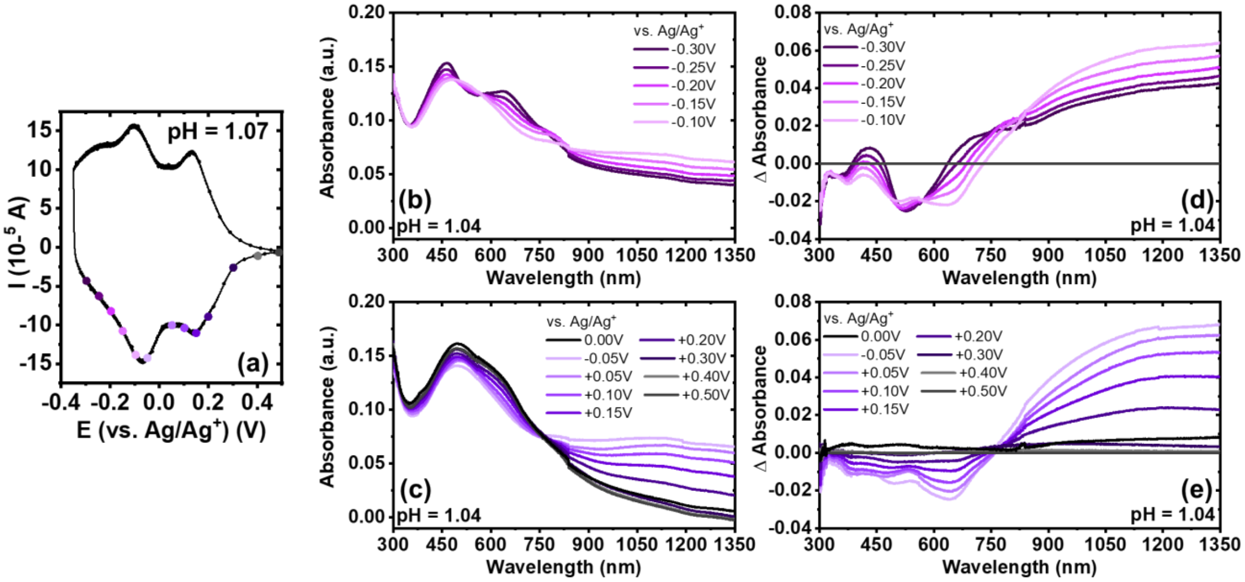

Figure S10. (a) Cyclic voltammogram of BBL-P thin films in aqueous KCl electrolyte of pH 1.07 collected at a scan rate of 25 mV/s; (b – c) UV-Vis-NIR optical absorption spectra under different potentials of BBL-P thin films in electrolyte of pH = 1.04; (d – e) Differential UV-Vis-NIR optical absorption spectra under different potentials of BBL-P thin films. The baseline spectrum was taken at +0.50V to de-dope the films prior to each doping step.

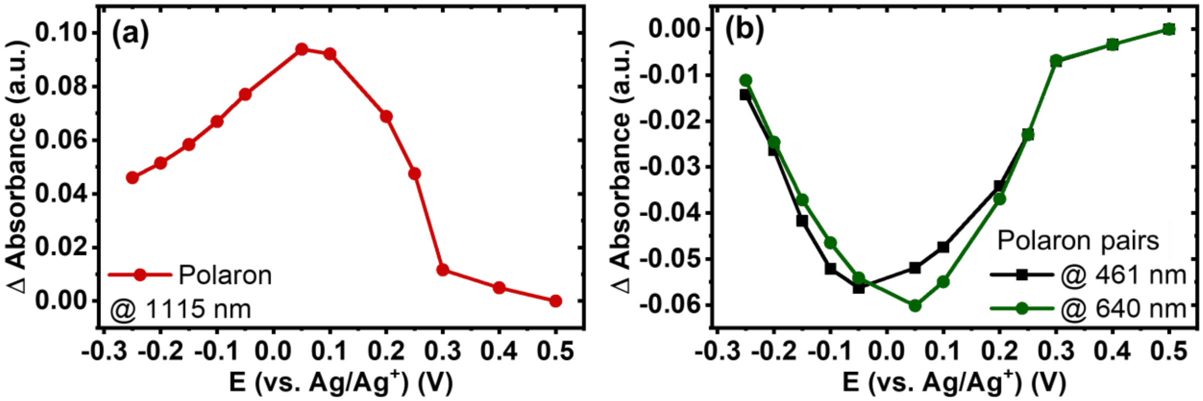

Figure S11. Dependence of differential absorbance of BBL-P in electrolyte of pH < 1 on doping potential at selected wavelength: (a) 1115 nm and (b) 461 nm and 640 nm.

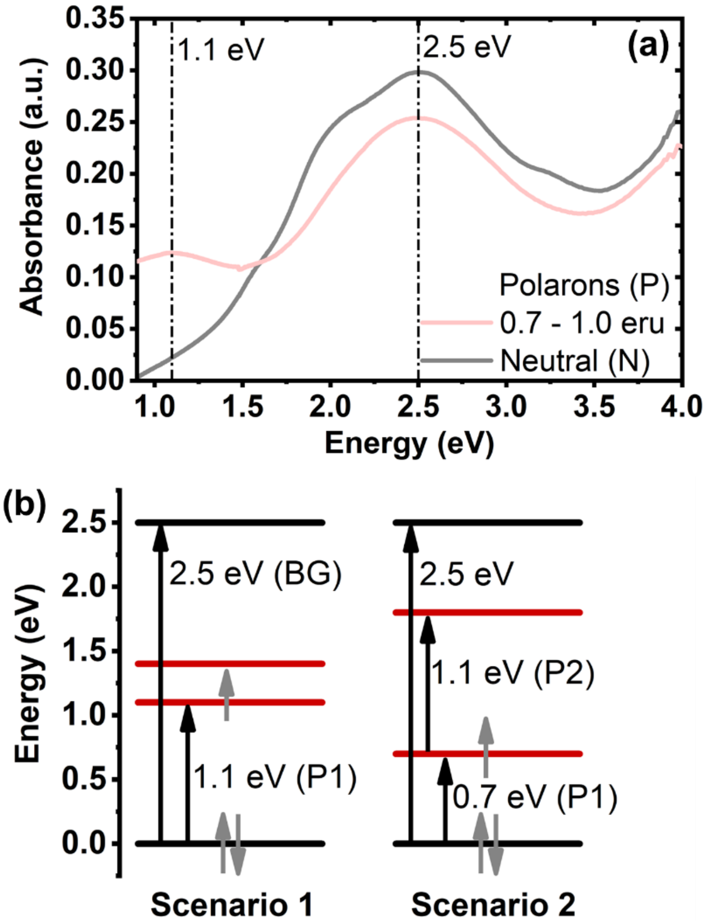

Figure S12. (a) Optical signatures of neutral BBL-P and polarons in doped BBL-P; (b) Possible electronic band structure of polarons in doped BBL-P.

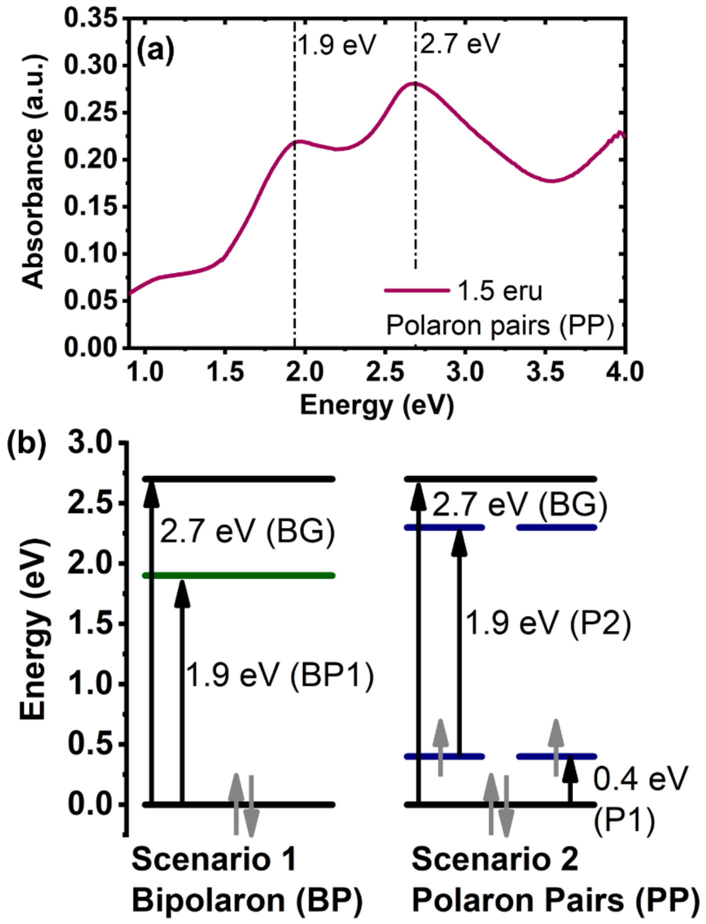

Figure S13. (a) Optical signatures of multiply charged species in doped BBL-P; (b) Possible electronic band structure of multiply charged species, bipolaron (BP) or polaron pairs (PP), in doped BBL-P.

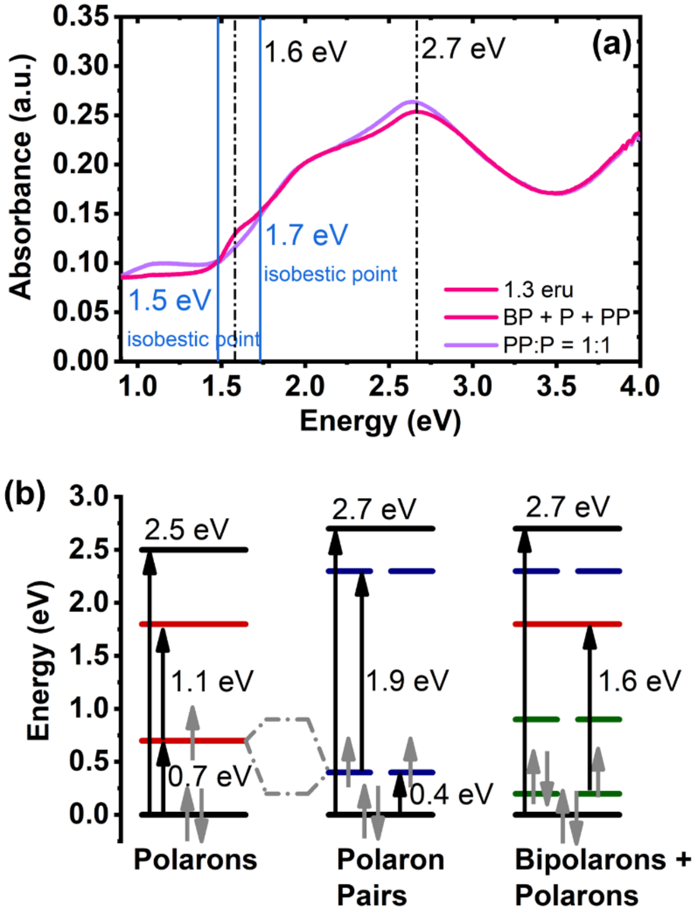

Figure S14. (a) Optical signatures of a mixture of singly charged and multiply charged species in doped BBL-P; (b) Hybridization between the bonding state of polarons and polaron pairs to yield bipolarons in doped BBL-P films.

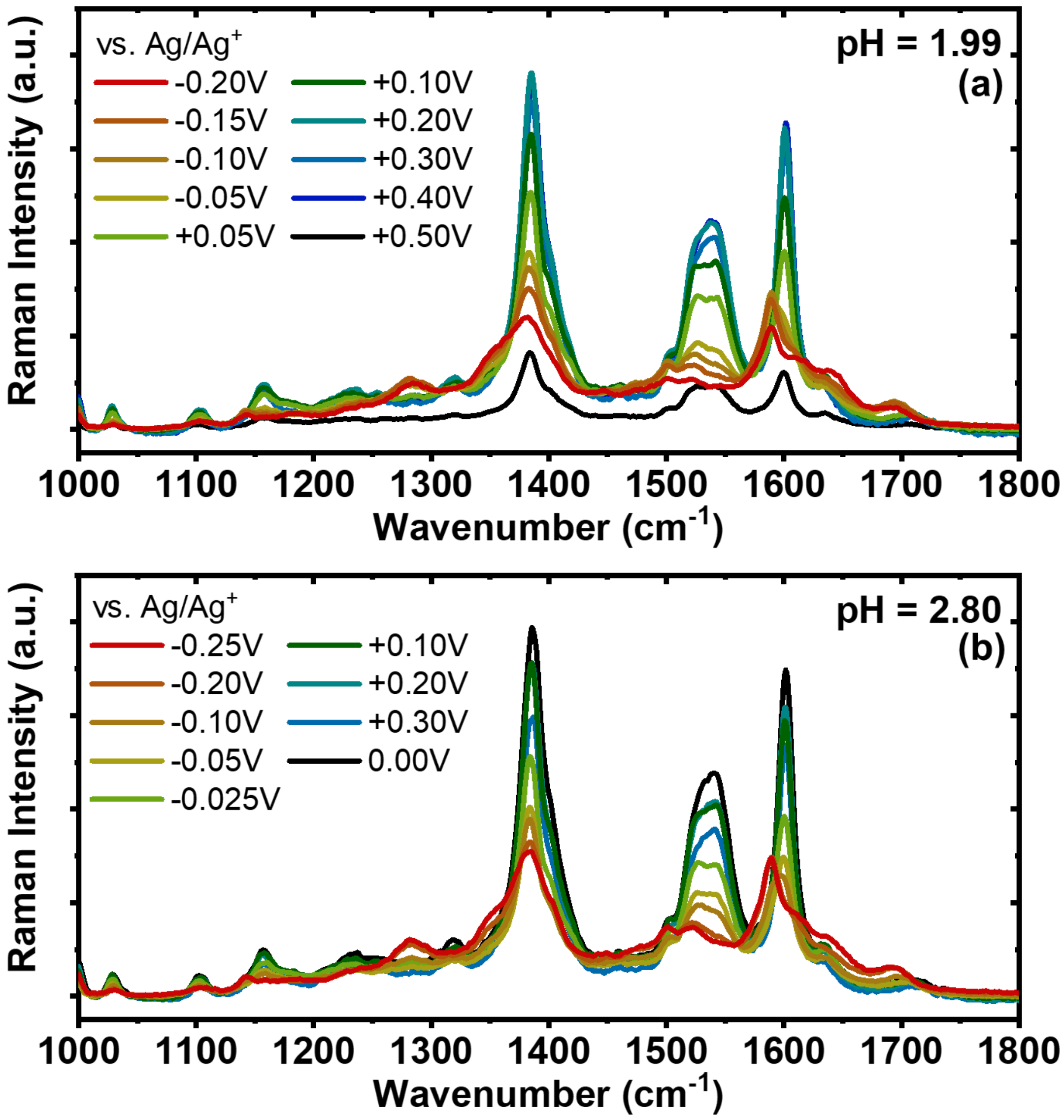

Figure S15. *In-operando* Raman spectra of BBL-P films collected in 0.1M $KCl_{(aq)}$ electrolyte of varying pH value: (a) pH = 1.99, and (b) pH = 2.80. Raman measurements were collected using 532 nm excitation with BBL-P films coated on gold covered glass substrates as the working electrode, Pt mesh as the counter electrode, Ag/AgCl pellet as reference electrode positioned away from the illuminated area.

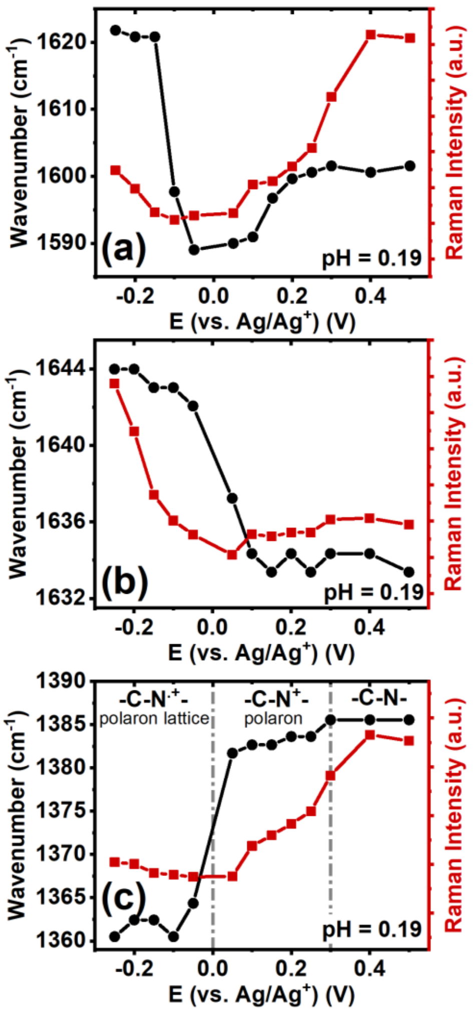

Figure S16. (a) Evolution of Raman frequency and Raman intensity associated with the C=C/C-C breathing of the naphthalene ring as a function of doping potential; (b) Evolution of Raman frequency and Raman intensity associated with the C=N vibrational mode as a function of doping potential; (c) Raman signatures of singly charged species (polaron) and multiply charged species (coupled polaron pairs).

Table S1. Quantification of Proton-Coupled Electron Transfer Processes of BBL Thin Films.

Wave	Slope[(a)] (mV/pH)	n_{H+}/n_{e-}[(b)]
I	-89.2 ± 5.2	1.5 ± 0.1
II	-99.8 ± 3.9	1.7 ± 0.1

[(a)]slope, average slope calculated from Pourbaix diagram (+/- one standard deviation);[(b)]n_{H+}/n_{e-}, number of protons per electrons transferred in each reduction event of BBL-P (+/- one standard deviation). Average values and standard deviations were calculated from 5 different samples.

Table S2. Summary of reduction potential of some redox active molecules.

Molecular Structure	E_1 (V) ($M \rightarrow M^{\cdot-}$)	E_2 (V) ($M^{\cdot-} \rightarrow M^{2-}$)	ΔE (V)	References
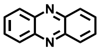	-1.4	-2.12	0.72	2, 3
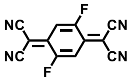	0.01[(a)]	-0.59[(a)]	0.60	4
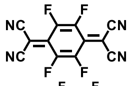	0.11[(a)]	-0.39[(a)]	0.50	4
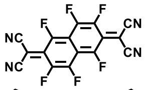	0.21[(a)]	-0.29[(a)]	0.50	4
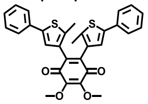	-0.58[(b)]	-1.23[(b)]	0.65	5
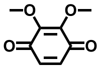	-0.66[(b)]	-1.37[(b)]	0.71	5
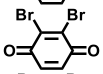	-0.24[(b)]	-0.97[(b)]	0.73	5
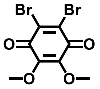	-0.35[(b)]	-1.1[(b)]	0.75	5
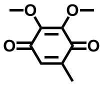	-0.69[(b)]	-1.34[(b)]	0.65	5
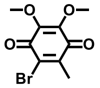	-0.54[(b)]	-1.27[(b)]	0.73	5
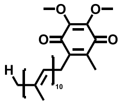	-0.77[(b)]	-1.39[(b)]	0.62	5
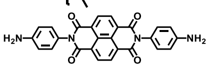	-0.96[(c)]	-1.43[(c)]	0.47	6
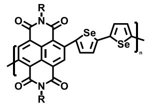	-0.85[(a)]	-1.29[(a)]	0.44	7

[(a)] Referenced vs. Ag/Ag^{+}.
[(b)] Referenced vs. SCE.
[(c)] Referenced vs. $Ag/AgClO_4$

Table S3. Density Functional Theory (DFT) Computed Atomic Charge Distribution of BBL-P Dimers in its Neutral State (Structure 1), Reduced at Carbonyl Oxygen State (Structure 1a), and Protonated Reduced State (Structure 2).

Atomic Position	Structure 1 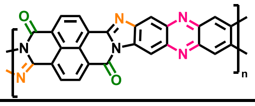	Structure 1a 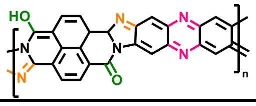	Structure 2b 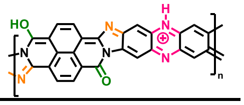
N46 (C=N, naphthalene)	-0.529	-0.549	-0.536
O48 (C=O, carbonyl)	-0.475	-0.558	-0.541
N45 (C=N, naphthalene)	-0.561	-0.588	-0.574
O47 (C=O, carbonyl)	-0.480	-0.557	-0.574
N81 (C=N, phenazine)	-0.562	-0.566	-0.520
N82 (C=N, phenazine)	-0.561	-0.574	-0.718
N24 (C=N, naphthalene)	-0.557	-0.598	-0.591
O23 (C=O, carbonyl)	-0.480	-0.544	-0.538
O18 (C=O, carbonyl)	-0.480	-0.547	-0.539
N17 (C=N, naphthalene)	-0.557	-0.600	-0.590
N69 (C=N, phenazine)	-0.561	-0.563	-0.715
N70 (C=N, phenazine)	-0.558	-0.564	-0.513

Table S4. Density Functional Theory (DFT) Computed Atomic Charge Distribution of BBL-P Dimers in its Neutral State (Structure 1), Protonated at Naphthalene Imine (Structure 3), Reduced Protonated at Naphthalene Imine (Structure 4a), and Reduced Double Protonated at Naphthalene Imine and Phenazine Imine (Structure 4b).

Atomic Position	Structure 1 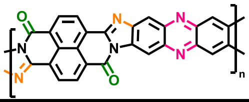	Structure 3 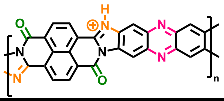	Structure 4a 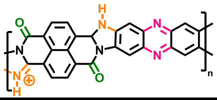	Structure 4b 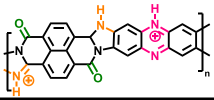
N46 (C=N, naphthalene)	-0.529	-0.503	-0.670	-0.644
O48 (C=O, carbonyl)	-0.475	-0.435	-0.455	-0.385
N45 (C=N, naphthalene)	-0.561	-0.738	-0.744	-0.721
O47 (C=O, carbonyl)	-0.480	-0.443	-0.464	-0.410
N81 (C=N, phenazine)	-0.562	-0.557	-0.569	-0.567
N82 (C=N, phenazine)	-0.561	-0.546	-0.567	-0.745
N24 (C=N, naphthalene)	-0.557	-0.537	-0.737	-0.726
O23 (C=O, carbonyl)	-0.480	-0.457	-0.466	-0.464
O18 (C=O, carbonyl)	-0.480	-0.429	-0.454	-0.478
N17 (C=N, naphthalene)	-0.557	-0.722	-0.735	-0.751
N69 (C=N, phenazine)	-0.561	-0.557	-0.560	-0.709
N70 (C=N, phenazine)	-0.558	-0.548	-0.549	-0.501

References

1. Frisch, M. J. *Gaussian 16*, Gaussian Inc.: Wallingford, CT, 2016.

2. Mugnier, Y.; Roullier, L.; Laviron, E. Reduction mechanism of phenazine in tetrahydrofuran. Influence of added proton donors. *Electrochimica Acta* **1991,** *36*, 803-809.

3. Laviron, E.; Roullier, L. Electrochemical reactions with protonations at equilibrium: Part IX. Comparison between the surface and heterogeneous electrochemical rate constants in the system phenazine/dihydrophenazine. *J. Electroanal. Chem. Interf. Electrochem.* **1983,** *157*, 7-18.

4. Kiefer, D.; Kroon, R.; Hofmann, A. I.; Sun, H.; Liu, X.; Giovannitti, A.; Stegerer, D.; Cano, A.; Hynynen, J.; Yu, L.; Zhang, Y.; Nai, D.; Harrelson, T. F.; Sommer, M.; Moule, A. J.; Kemerink, M.; Marder, S. R.; McCulloch, I.; Fahlman, M.; Fabiano, S.; Mueller, C. Double doping of conjugated polymers with monomer molecular dopants. *Nat. Mater.* **2019,** *18*, 149-155.

5. Simeth, N. A.; Kneuttinger, A. C.; Sterner, R.; König, B. Photochromic coenzyme Q derivatives: switching redox potentials with light. *Chem. Sci.* **2017,** *8*, 6474-6483.
6. DeBlase, C. R.; Hernández-Burgos, K.; Rotter, J. M.; Fortman, D. J.; dos S. Abreu, D.; Timm, R. A.; Diógenes, I. C. N.; Kubota, L. T.; Abruña, H. D.; Dichtel, W. R. Cation-Dependent Stabilization of Electrogenerated Naphthalene Diimide Dianions in Porous Polymer Thin Films and Their Application to Electrical Energy Storage. *Angew. Chem. Int. Ed.* **2015,** *54*, 13225-13229.
7. Tran, D. K.; Robitaille, A.; Hai, I. J.; Ding, X.; Kuzuhara, D.; Koganezawa, T.; Chiu, Y.-C.; Leclerc, M.; Jenekhe, S. A. Elucidating the impact of molecular weight on morphology, charge transport, photophysics and performance of all-polymer solar cells. *J. Mater. Chem. A* **2020,** *8*, 21070-21083.
